# How Organisational and Socio-Cultural Contexts Shape Healthcare Workers’ Intrinsic, Prosocial, and Public Service Motivation in Africa: A Scoping Review

**DOI:** 10.34172/ijhpm.8861

**Published:** 2025-10-04

**Authors:** Djibrine Diallo, Bruno Marchal, Zakaria Belrhiti

**Affiliations:** ^1^Mohammed VI International School of Public Health, Mohammed VI University of Sciences and Health, Casablanca, Morocco.; ^2^Laboratory of Public Health and Management Department, Mohammed VI Center for Research & Innovation, Rabat, Morocco.; ^3^Department of Public Health, Institute of Tropical Medicine, Antwerp, Belgium.

**Keywords:** Contexts, Public Service Motivation, Health Workers, Theories, Africa

## Abstract

**Background:**

In Africa, the poor quality of care is often attributed to a lack of motivated health workers (HWs). Most reforms implemented in African health systems rely on performance-based financial incentives. Evidence suggests that financial incentives may have adverse effects, such as crowding out autonomous forms of motivation, including intrinsic, prosocial, and public service motivation (PSM). We aim to map conceptual definitions of autonomous motivation and unpack the relationship between context and societal culture in shaping the motivation of HW in Africa.

**Methods:**

Following guidelines from Arksey and O’Malley, we conducted a scoping review of peer-reviewed publications from 1990 to 2024 using the databases (Web of Science, Scopus, Google Scholar, and PubMed). We used the Intervention-Context-Actor-Mechanism-Outcome (ICAMO) heuristic to identify plausible causal pathways linking context and societal culture to HW motivation.

**Results:**

Scholars defined PSM as a complex dynamic process that stimulates individuals to carry out self-altruistic and prosocial behaviours. Our review showed that autonomous motivation is sensitive to context. Enabling conditions include a positive work environment, community appreciation, and local context. Our review suggests a form of intrinsic motivation (IM) for HW in Africa that may be rooted in collectivistic values, such as the willingness to serve the community to which they belong. When HW perceived a lack of belonging to the community they serve, they reported being disregarded, which reduced their sense of self-efficacy, self-esteem, and trust in their community relationships.

**Conclusion:**

This review highlights how context and societal culture can reinforce the trust relationship between HW and communities, thereby increasing HWs’ motivation by enhancing their perceived self-efficacy and autonomy. Our findings suggest that exploring the role of decentralisation, trust relationships, and self-efficacy in expressing autonomous motivations are research priorities.

## Introduction

 Health workers’ (HWs’) motivation is an essential driver of HWs’ performance and quality of care, and it is a central element in health system performance.^1-3^ However, in low- and middle-income countries (LMICs), especially within African healthcare systems, several challenges such as staff shortage, resource scarcity, poor working conditions, inequitable distribution of HW across geographic and healthcare settings, lack of appropriate management and compensation systems, and low motivation levels,^1,3^ often hamper the performance of health services and hinder the implementation of proven effective health system reforms.^4^

 In Africa, HW often experience low motivation due to organisational factors such as a lack of staff, which adversely affects job satisfaction and performance.^5,6^ Similarly, inadequate managerial practices, such as weak supervisory support, a lack of transparent promotion systems, inadequate training and a lack of equipment, are critical demotivators for HW, negatively impacting their performance to deliver quality of care.^7,8^ Socio-cultural dynamics and community expectations further compound these systemic challenges.^9,10^

 In light of these challenges, a deeper understanding of factors that improve and sustain HWs’ motivation to perform and deliver high-quality care, is important to foster sustainability, resilience and performance of health systems in Africa.^11,12^

 Motivation is the individual’s willingness to sustain efforts toward organisational goals.^3^ It should be noted that several motivation theories^13^ relevant to human resource management (HRM) have been developed across disciplines, and the most conceptualised in the healthcare field^14^ are prosocial motivation (PM) from organisational behaviour,^15^ public service motivation (PSM)^1,16,17^ from public administration,^17^ and self-determination theory (SDT) from psychology.^18^

 The academic management literature distinguishes between controlled or extrinsic motivation and autonomous motivation, which includes intrinsic motivation (IM), PM, and PSM.^18-20^

 IM is defined as the satisfaction the work provides, whereas extrinsic motivation refers to an external locus of control.^21^ In healthcare, IM is often positively associated with job satisfaction, individual and organisational performance, and quality of care.^10,22^ Intrinsically motivated HW in the public sector often perform in the best interest of patients and the public.^23,24^ In some African healthcare settings, like Ethiopia and Morocco, HWs have described IM as the inherent satisfaction derived from providing care itself, despite poor working conditions, because it gave them a sense of personal purpose and professional pride.^23,25^

 PM “*is defined as the desire to perform actions in ways that are beneficial to other individuals* or the public at large.”^15^ HW with prosocial preferences are highly motivated to provide quality care.^26^ In African health systems marked by significant disparities in HW distribution between urban and rural areas, along with other issues such as delayed salaries, prosocial values have been positively associated with the willingness to work in underserved rural areas.^27,28^ In Morocco, HW expressed their PM in religious or spiritual terms, describing public service delivery as a moral duty rooted in their beliefs, shaping prosocial behaviour.^29^ PM is considered a defining feature of PSM.^20^

 PSM is “*an individual’s predisposition to respond to motives related primarily or solely to public institutions and *organisations.”^30^ PSM was first described as a driver of motivation of public servants by Perry and Wise in North America^31^ and later in Europe and the UK.^32^ In Europe, the most commonly used definition of PSM was developed by Vandenabeele, who defined PSM as “*the belief, values, and attitudes that go beyond personal and organisational interest, which relate to the interest of a larger political entity and motivate individuals to act accordingly when appropriate.”*^33^ The nature and mission of public institutions, as well as individual beliefs, values and attitudes, are all factors shaping PSM.^34^ PSM includes four dimensions: Attraction to the public interest, participation in the processes of political decisions, self-sacrifice, compassion, and commitment to public values.^35^ *Attraction to the public interest *refers to internal satisfaction or enjoyment from serving the public or participating in public policy-making.^30^ *Self-sacrifice *is defined as forgoing personal interests to serve the public interest.^30^ Compassion refers to emotional empathy towards human beings, whereas commitment to public values is oriented towards civic duty and ethical government responsibility.^20,34,35^

 In practice, HW often commit to public values and display affective compassion towards patients.^29,36,37^ HW describe their profession not merely as employment but as a “calling,” especially when working in challenging conditions.^29^ The *compassion* and *self-sacrifice* aspect of PSM is illustrated by how HW go beyond their roles to ensure patient welfare, including paying for patients’ medication from their own pockets.^29^

 These autonomous forms of motivation have in common the willingness of individuals to contribute to public service, to focus on doing good to others and to shape the well-being of society.^20,33^

 In healthcare, the terms IM, PM and PSM are often discussed in referring to intrinsic drivers as the primary construct for explaining altruistic and duty-driven behaviours,^20,38,39^ but are not clearly used interchangeably.^20,40^ To address this gap in definitional clarity, we will build on Vandenabeele and colleagues’ conceptual framework and in line with the work of Perry and Wise, and Ritz et al.^20,41^ We will be using ‘PSM’ as a synonym for all three forms of autonomous motivation, whereby the latter is defined as all the forces that motivate individuals to selflessly help others for the common good without expectation of any reward.^20^

 In Africa, recent health workforce reforms, inspired by new public management, are often based on introducing financial incentives.^10,42-44^ However, recent evidence suggests that such performance-based financing reforms, particularly financial incentives, have often unintended consequences, including crowding out the autonomous forms of HWs’ motivation and poorly sustained organisational commitment of HW if financial incentives are suspended.^42,45^ Financial incentives may undermine HWs’ compassion for patients when the emphasis is placed on achieving the goal of financial gain rather than on patient-centred care and the public interest.^46^ Such approaches based on incentives alone are insufficient in the African healthcare context, and scholars highlight the critical role of autonomous motivation, such as IM, in sustaining and improving motivation and quality of care among HW.^46,47^

 While scholars argued that PSM is an internationally valid empirical concept,^31^ they also emphasized that cultural differences may explain variations in the meanings and expression of PSM across different cultures.^16^ Evidence indeed suggests that the expression of PSM might be shaped by cultural values, and religious, and spiritual beliefs, with spirituality shaping altruistic behaviours and HW viewing patient care as a spiritual duty.^29,48,49^ Within some African healthcare settings, HWs’ commitment and willingness to perform care were positively correlated with religious and spiritual duty, and healthcare decisions are rooted in divine purposes, highlighting the importance of religious and spiritual beliefs.^50^

 The PSM framework was predominantly developed and tested in Western individualistic societies, which may not fully capture the collectivist values, social norms, and spiritual beliefs that shape most professional behaviour in African societies.^16,51,52^ There is scarce evidence regarding the contextual validity of PSM in African healthcare settings and its congruence with the local context and societal culture, highlighting the need for conceptual adaptation and empirical synthesis to align motivational strategies with African local cultural contexts.^16,29^ Few studies have addressed the role of context, including societal culture, in shaping the expression of HWs’ motivations.^41,53-55^ Some exceptions are studies by Tamba et al and Bawole et al.^16,56^ We refer to contextual elements as factors or variables beyond the intervention, such as the economic, social, and political environment (eg, resources, local culture, laws, regulations, etc), which may shape HWs’ motivation.^57,58^

 Understanding how the societal culture or contextual elements shape the expression of PSM by HW is essential for the design of suitable motivational strategies in African healthcare settings.^59^ Through the literature research, to our best knowledge, there are no systematic reviews about how the contextual elements and societal culture affect HWs’ autonomous motivation in African healthcare settings.^16^ Addressing this gap will inform suitable motivational strategies in Africa that align with local values and socio-cultural expectations.^16,29^

 With this study, we set out to review the evidence on the role of context and societal culture in shaping the expression of PSM among African HW.

## Methods

 We carried out a scoping review following the guidance of Arksey and O’Malley and Levac et al.^60,61^

###  Stage 1: Identifying the Research Question

 We set out to identify evidence on the relationship between societal culture, contextual elements and the expression of PSM in African healthcare settings.

 The research questions include:

1. How is the notion of PSM defined, theorised, conceptualised, and operationalised in African healthcare settings?
2. What underlying processes link the societal culture and contextual elements to the expression of PSM among HW in African healthcare settings?

###  Stage 2: Identifying Relevant Studies

 We included peer-reviewed articles, theses, books, and book chapters published between 1990 and 2024 that specifically mention the concept of “PSM,” “prosocial motivation,” or “intrinsic motivation” in the title and abstract. All study types were included. African countries used in our search strategy were based on the World Bank’s definition of African LMIC (See our search strategy in Supplementary file 1, Table S1 and Box 1).^62^ We used the Perry and Wise definition of PSM to identify papers covering autonomous, prosocial, and IM. Only peer-reviewed literature was included. We excluded the grey literature, commentaries, and conference proceedings. Studies carried out outside Africa were also excluded.

**Box 1. Population, Concept, and Context Framework**

***Population:** Health Workers **AND Concept 1:*** Societal culture OR dimension “masculinity” OR “femininity,” “collectivism” OR “individualism” OR “power distance,” OR “indulgence OR restraint,” “short-term/long-term orientation” OR “uncertainty avoidance.” AND ***Concept 2:*** Public service motivation, intrinsic or prosocial form of motivation (intrinsic and prosocial) and health professionals. AND **Context:** Healthcare settings in Africa.

###  Stage 3: Study Selection

 Our search strategy was formulated using the *Population, Concept, and Context framework*^63^ (See Supplementary file 1, Table S1).

 An example of a search string we used in the Web of Science database is: (“public service motivation” OR “prosocial motivation” OR “intrinsic motivation”) AND (“societal culture” OR “collectivism” OR “individualism” OR “indulgence OR restraint” OR “uncertainty avoidance” OR “power distance” OR “short-term/long-term orientation” OR “uncertainty avoidance”) AND (“health worker”) AND (“healthcare settings”). We carried out an additional manual search using citation tracking and the snowballing technique. The searches were carried out on May 4, 2022 and subsequently updated on February 1, 2024 (See Supplementary file 1, Table S1). We searched PubMed, Scopus, Web of Science, and Google Scholar databases, as they cover relevant literature in social science, health systems research and public organisation, aligning with the interdisciplinary scope of our review.

 Our search retrieved 24 067 publications. After removing 3607 duplicates with the assistance of a citation and reference manager (Zotero), we screened the titles and abstracts of the remaining 20 460 papers. Based on the inclusion and exclusion criteria, 18 992 papers were excluded. We then extracted 1468 papers for abstract and result screening by two reviewers (DD and ZB). Inter-rater agreement between the two reviewers was assessed using Cohen’s kappa statistic.^64^ The observed agreement was 97.3%, with an expected agreement based on classification based on marginal distribution of 91%. The resultant kappa value was 0.696 (Standard Error = 0.047), indicating good agreement according to Altman’s classification.^64^ The 95% confidence interval ranged from 0.603% to 0.789%, supporting the reliability of the reviewers’ judgements and the findings (See Supplementary file 2, Table S2). We limited the included studies to those published in French, English, and Arabic, as these are the working languages of the authors, allowing for accurate screening and data extraction in accordance with methodological guidance that recommends aligning language criteria with the authors’ capacity.^65^ Only 18 papers were included in this review (See Supplementary file 3, Figure S1).

###  Stage 4: Charting the Data

 Data were extracted into a data chart using Microsoft Excel software. The data extraction form is presented in Box 2. Data charting includes the general characteristics of included studies (author, country, date of publication, study types), intervention characteristics (financial incentive policy and managerial practices to improve HWs’ motivation), the contextual conditions and mechanisms of changes (understood as explanatory accounts, of the relationship between IM, PM or PSM, the societal culture) and reported outcomes of the financial incentive and managerial practices on HWs’ motivation. We followed reporting guidelines for scoping reviews (See Supplementary file 4, Table S3).^66^ Thematic analysis was conducted using Dedoose, a qualitative data analysis software, to code and synthesise key results using the data charting forms in Box 2.

**Box 2. Data Extraction Form**

- Author, date, country of publication, study type
- Conceptual definitions of IM, PM, or PSM
- Contextual conditions: (1) Societal cultural dimensions according to Hofstede’s Cultural and gender norms, values, practices, beliefs (See Supplementary file 4, Tables S3 and S4), (2) Other contextual elements (social, economic, and policy factors)
- Characteristics of motivational intervention strategies
- Reported outcomes (individual, team, and organisational)
- Explanatory accounts (theories) of the relationship between HWs’ motivation, and the societal culture or gender norms, values, practices and beliefs or contextual elements
- Research gaps, and methodological and practical implications

-----------------

Abbreviations: PSM, public service motivation; PM, prosocial motivation; IM, intrinsic motivation; HWs, health workers.

###  Stage 5: Collating, Summarizing and Reporting the Results Coding and Thematic Analysis

 To code the different types of motivation (IM, PM, and PSM), we used Vandenabeele and colleagues’ conceptual framework that distinguishes between IM, PM, and PSM based on the nature of their intended recipients.^20^ To code the different dimensions of societal culture, we used Hofstede’s^67^ operational definitions of cultural subdimensions (power distance, uncertainty avoidance, individualism/collectivism, masculinity, feminism, and long-term and short-term orientation, indulgence versus restraint) (See Supplementary file 5, Tables S4 and S5).

 We used a qualitative evidence synthesis approach, guided by thematic analysis to inductively code and categorise contextual elements and sociocultural factors shaping HWs’ motivation in the included studies, following best practices in concept-driven coding.^68,69^

 We used concept-driven coding with Dedoose software (See coding list in Supplementary file 6, Box S1). The included studies were coded manually and then organised and completed in Dedoose. The coding process involved data categorisation, focusing on key themes shaping HWs’ motivation. We also used a multi-layered ecological framework^70^ that includes, at the macro level, the government policy and interventions, cultural and gender norms, values, practices, and beliefs; at the meso level, the healthcare facilities and institutions and at the micro level, the role of the interaction between HW and patients. The inductive approach was used to identify underlying mechanisms across 18 included studies. The coding process included iterative cycles of refinement and validation among authors to enhance analytical credibility.^71,72^ This iterative approach and collaborative process included regular discussion, reflexivity and revisions to ensure that the findings were grounded in the included studies (Supplementary file 7, Box S2) and aligned with best practices in thematic analysis, thereby strengthening the nuanced interpretation and trustworthiness of the findings.^71,72^

 We synthesised plausible causal relationships using the Intervention-Context-Actor-Mechanism-Outcome (ICAMO) configuration.^73,74^ In realist inquiry, ICAMO serves as an analytic heuristic to describe the causal chain that explains how an intervention leads to an outcome by triggering specific mechanisms for different actors in different contexts.^73^ We refer to the mechanism as the underlying processes triggered by an intervention in a specific context.^73^ We adopted the taxonomy of mechanisms developed by Astbury and Leeuw, who grouped mechanisms into three categories. Situational mechanisms operate at the macro-to-micro level and show how specific social situations shape individual actors’ beliefs and desires.^53^ Action-formation mechanisms operate at the micro-to-micro level and explain how individual choices and actions are influenced by desires and beliefs.^73^ Transformational mechanisms operate at the micro-to-macro level, showing how individuals’ actions and interactions generate macro-level outcomes.^73^ Community context, policy context, gender and cultural norms, values, practices, and beliefs were coded as the sociocultural and contextual elements (See Supplementary file 6, Box S1).

## Results

 We first present an overview of the characteristics of the included studies. We then summarise how PSM and its related concepts (PM and IM) are defined and operationalised. We end by presenting the underlying processes that relate to the societal culture, contextual elements, and the expression of PSM among HW in African healthcare settings and suggest some practical recommendations.

###  Characteristics of Included Studies

 Most studies were published in East Africa (Ethiopia, Kenya, Rwanda, Somalia, South Africa, Tanzania, Uganda, and Zimbabwe); North Africa (Algeria, Egypt, and Morocco); and West Africa (Burkina Faso, Ghana, and Sierra Leone). Most studies were carried out in Tanzania (n = 4), Ghana (n = 3) and Morocco (n = 3). Other studies were multicentric, covering several countries. These included Uganda, Sierra Leone, and Zimbabwe (n = 1)*, Burkina Faso, Ghana, and Tanzania (n = 1)*, Ethiopia and Rwanda (n = 1)*, Algeria (n = 1), Egypt (n = 1), Kenya (n = 1), Somalia (n = 1), and South Africa (n = 1). Asterisks indicate international studies conducted across two or more countries (See Supplementary file 8, Figure S2). We identified three international studies that addressed PSM and IM of HWs in three African settings (See Supplementary file 8, Figure S2). Frequency of countries mentioned in the publications.

 Our study included 18 primary studies. The majority of these studies were qualitative and exploratory, and all of them were case studies based on interviews and focus group discussions (56 %, n = 10), with only 33% (n = 6) using quantitative descriptive approaches (questionnaires), and 11% (n = 2) of studies using mixed methods design (interviews and questionnaires as shown in Table). Quantitative and mixed-methods studies assessed IM, PM, and PSM using survey instruments, with some reporting reliability measures such as Cronbach’s alpha or validated PSM scales (See Supplementary file 9, Table S6).

**Table:** General Characteristics of Included Studies

**Authors**	**Year **	** Methods **	**Data Collection Methods**	**Sample Size**	**Study Population**
Hopwood et al^75^	2023	Qualitative	Interview and FGD	13	Mental health nurses and psychiatrists
Adjei-Mensah^76^	2023	Quantitative	Survey	372	Healthcare professionals
Sheikh et al^77^	2023	Quantitative	Questionnaire	220	Nurses, midwives, paediatricians, and gynaecologists
Rim et al^78^	2021	Quantitative	Questionnaire	48	Nurses
Belrhiti et al^50^	2020	Qualitative	Interview and FGD	32	Nurses and physicians
Belrhiti et al^25^	2020	Qualitative	Interview and FGD	146	Nurses and physicians
Belrhiti et al^29^	2019	Qualitative	Interview	146	Nurses and physicians
Witter et al^79^	2018	Qualitative	Interview	103	Nurses, midwives, and medical doctors
Brenya et al^80^	2016	Mixed method	Interview and questionnaire	21	Physicians
Gould-Williams et al^81^	2015	Quantitative	Questionnaire	340	Nurses and physicians
Okuga et al^82^	2015	Qualitative	Interview	32	Nurses and midwives
Ojakaa et al^83^	2014	Quantitative	Questionnaire	404	Nurses and physicians
Prytherch et al^44^	2013	Qualitative	Interview	25	Nurses, midwives, and medical doctors
Prytherch et al^84^	2012	Qualitative	Interview	105	Nurses, midwives, and medical doctors
Songstad et al^85^	2011	Qualitative	Interview and FGD	33	Nurses and physicians
Luboga et al^86^	2011	Mixed methods	Interview and questionnaire	112	Physicians
Serneels et al^87^	2010	Quantitative	Questionnaire	412	Nurses and physicians students
Stringhini et al^88^	2009	Qualitative	FGD	64	Nurses, midwives, and medical doctors

Abbreviation: FGD, Focus Group discussion.

###  Definition and Measurement of Public Service Motivation 

 Five studies out of 18 explicitly refer to the definition by Vandenabeele,^25,29,50,80,81^ who defines PSM as “*the beliefs, values and attitudes that go beyond self-interest and organisational interest, that concern the interest of a larger political entity, and that motivate individuals to act accordingly whenever appropriate.” *Some scholars have focused on specific dimensions of PSM, such as attraction to public policy and the role of organisational context (person-organisation fit) in Egypt,^81^ while in Morocco, compassion and self-sacrifice were considered key defining dimensions of PSM among HW.^29,50^ Most quantitative included studies referred to Perry’s measurement scale^30^ to measure and examine factors impacting PSM in the workplace.

 Other scholars used the Deci and Ryan definition of IM as the energisation, direction, and persistence of behaviour among HW.^44,76,77,84,86,87^ All papers refer to motivation as a complex process that involves a set of factors, including work environment, community appreciation, and social context.^3,20^

**Figure 1:**
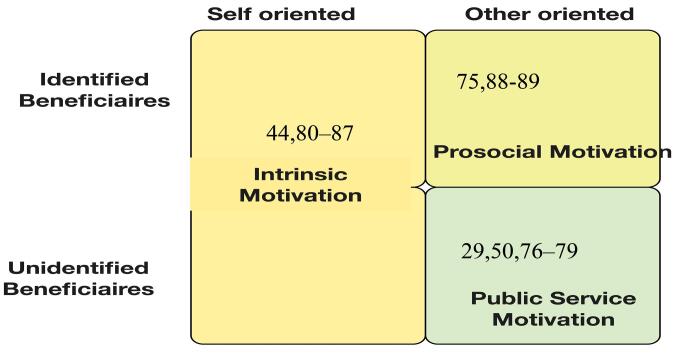
Classification of Studies According to the Conceptual Clarification of Vandenabeele.

 Authors argue that the motivation of HW is a dynamic psychological process encompassing both intrinsic and extrinsic components.^21,39,54^ It includes IM driven by personal satisfaction, PM focused on helping identifiable individuals, and PSM oriented towards serving the public without direct identification of recipients, as shown in Figure 1.^20^

 Only two studies have provided definitions of culture using the Hofstede framework.^86,89^ In Sierra Leone, culture was defined as the shared beliefs, values, customs, behaviours, and practices that characterise a particular group or society.^75^ Others referred to Franco^3^ and Schwartz, who defined culture as “*the common ways of thinking, feeling, and acting that are shared among members of a society or related social group.”*^44^

###  How Societal Culture Shapes the Expression of PSM 

 Scholars from included studies highlighted the importance of specific cultural beliefs, such as “uncertainty avoidance, and anxiety” among physicians,^67,86^ power distance,^90^ and informal payment in Tanzania,^88^ which in turn affect their PSM and motivation to deliver quality care.

 Our review findings suggest that the expression of PSM can be shaped by cultural norms and shared values, such as a strong attachment to family relationships, loyalty, and attachments to spiritual and religious beliefs (Morocco,^29^ Somalia,^77^ and Tanzania^84^). We summarised the relationship between societal culture, contextual elements, HW, and PSM using the ICAMO configurations as a heuristic framework. Each ICAMO addresses different underlying mechanisms at the macro, meso, and micro levels.

###  ICAMO Configuration Analysis at the Macro-Level 

####  How Unfavourable Socio-Cultural and Political Contexts May Hinder PSM in Healthcare Settings 

 (I) Poor HRM management, lack of regulations, and inappropriate policy reform, (C) which fail to respond to low incomes and high living costs, might be the primary causes of the widespread corruption and dishonesty, which have been made worse by the large power distance of the government, (A) HW (nurses and doctors) turned to informal payments to supplement their income, (M) which conflicted with their values, triggering^75^ (situational mechanism)^73^ *feelings of guilt and *reduced *self-esteem and led them to be controlled by the patient. It contributed to negative reciprocity* from HW. *Perceived organisational politics* (eg, Clientelism and nepotism) lead to psychological distress and low organisational commitment, which might impact the productivity and fairness of HW. Contribute to widespread corruption,^75^ clientelism, nepotism, and organisational politics^50^ reduced IM, PSM, and *individual performance *(O).

####  The Demotivating Factors of Decentralisation in Mental Health Services

 (I) The decentralisation of nurse-led mental health services was introduced to enhance service access and empower (A) mental health nurses and psychiatrists. (C) The context into which the programme was introduced is characterised by a lack of explicit prioritisation of mental health programmes. This, combined with high power distance and high uncertainty avoidance due to bureaucratic hierarchy and lack of recognition, (M) decentralisation perceived not as empowerment leads to decreased perceived organisational support (delay in resource allocation), (O) which may contribute to job dissatisfaction, as a result, reduced nurses’ and psychiatrists’ IM, undermining the intended benefits of the decentralisation reform.^75^

###  ICAMO Configuration Analysis at the Macro- and Meso-Level:

####  The Role of Political and Organisational Factors on Health Workers’ Motivation

 (I) Inappropriate political influence in the healthcare system manifested in local politicians’ intimidation methods and district-level politicians’ interference in hospital decision-making, (C) within a context marked by strong uncertainty avoidance in Uganda, Tanzania, and Ghana,^67^ characterized by a lack of transparency in government regulations and decision-making regarding HRM, promotion, recognition, and allowances (M) may significantly shape (A) physicians’ and nurses’ perceptions of unfairness and procedural injustice in their working conditions and such perceptions may (O) contribute to job dissatisfaction and reduced organisational commitment, which may negatively impact IM and PSM levels and lead to brain drain.^76,85,86^

 (C) In a work environment influenced by political factors, (I) through nepotism and clientelism (over managerial decisions), physicians and nurses (A) may (M) *perceive a lack of autonomy at work and perceive organisational politics*. (O) Then, this results in job dissatisfaction, which leads to decreased PSM among HW.^50,76^

####  The Role of Bureaucracy on Health Workers’ Motivation

 (I) Government centralisation of power both at the macro level from the Ministry of Health and at the meso-level through over-controlling district managers, (C) characterised by a lack of transparency in salary categorisation and limited access to information about working conditions, which restricts nurses’ and physicians’ freedom to make informed comparisons and decisions. (A) Nurses and physicians interviewed acknowledged that national legislation restricts district-level decisions on the availability of information on salary scales and allowances, (O) which might lead to disparities, biased selection processes for training opportunities, (M) and a *perceived lack of procedural justice and autonomy in decision-making*, (O) resulting in demoralisation and discouragement and may reduce nurses’ and physicians’ IM.^85^

 (C) In an environment characterised by power distance (strong power distance, eg, Ghana and Morocco), and manifested through (I) mechanical bureaucracies such as top-down rules, regulations, and poor managerial practices, lack of participative decision-making and poor recognition of (A) nurses and clinicians’ efforts at the Meso-level, (M) the l*ack of perceived autonomy of *nurses and clinicians and poor work environment (I) is triggered when managers make decisions that may directly affect their work without involving them, coupled with ineffective communication strategies, (O) leading to psychological distress and job dissatisfaction and may reduce individual performance and IM of nurses and clinicians.^50,85,86^

####  The Role of Poor HRM Practices on Health Workers’ Motivation

 (C) In the context characterised by inappropriate promotion policies at both the central and district levels, manifested through (I) the lack of recognition of working experience. (C) This working environment where there is a lack of transparency in decision-making regarding HRM, promotion, recognition, and allowances may lead qualified (A) nurses and physicians (M) to perceive* procedural injustice* and perceive themselves as inequitably treated, (O) which may result in job dissatisfaction undermines IM and reduced individual performance.^85,86^

####  The Role of Masculinity and Policy Gaps on Health Workers’ Motivation

 (C) In patriarchal culture that prevails in Somalian healthcare settings, hinders female gynaecologists’ involvement in decision-making and keeps female gynaecologists in lower professional positions than male gynaecologists.^77^ (I) This is made worse by the lack of appropriate policy reforms or organisational policies to enforce equal treatment and career advancement opportunities. (A) Female gynaecologist may perceive these factors as obstacles to career advancement, and may find it difficult to deal with hostile conditions and (M) may feel less organisational supported, which may discourage them to perform well and undermine IM.^77^ *Culturally non-sensitive healthcare services* (O) may lead to a bad working environment, (M) making them feel unrecognised and marginalised.^77^

###  ICAMO Configuration at the Meso-Level: Value Incongruence on HWs’ Motivation in Collectivist Societies 

####  Value Congruence is the Degree of Alignment Between Health Worker and Organisational Values

 (I) In Poor management practices “characterised by clientelism and nepotism,” (C) in a collectivistic society where co-exists strong family education related to the respect of societal norms and values, such as not accepting “bribes,” and strong religious beliefs, such as expected rewards from “God.”^29,50^ (A) Nurses, physicians, and clinical officers experienced (M) “value incongruence”; they argued that the management’s nepotism and clientelism went against their values. This led to the emergence (M) of mistrust and perceived politics inside the organisation.^29,50^ (O) Lower commitment at the individual level and decreasing PSM.

###  ICAMO Configuration at the Micro-level: Community Appreciation as Drivers of Health Workers’ Motivation

 (C) In a context shaped by collectivism (*People are born into extended families or clans which protect them in exchange for loyalty).*^67^ (A) Nurses and clinical officers often find meaning in (I) their interaction with the patients. In such environments, nurses and clinical officers experience “value congruence,” HW showed shared values (M) for *recognition and support of HW and community appreciation*. As one HW noted, “this is my reward because I see that I can help these people.”^84^ The influence of loyalty to the family is significant. This intrinsic driver and affirmation (O) may contribute to enhancing job satisfaction, ultimately leading to increased IM among HW.

 (C) In a challenging environment where co-existing limited resources and difficult working conditions, (I) *the support and appreciation by the community may* enhance the (A) nurses’ and physicians’ (M) sense of value and professional identity and make *them feel recognised by the community*, (O) leading to higher job satisfaction and commitment, a supportive working environment and may increase PSM, IM, and PM.^44,83,86,87^

 (C) In a collectivistic society, where religion (eg, Muslim and Adventist) is highly valued, it may foster a sense of community among (A) nurses, medical doctors, where family relationships are highly valued. (I) This sense of belonging may shape the behaviours of HW, (M) boost their level of compassion and self-sacrifice, and give them a sense of support and recognition from the community, (O) all of which may increase their job satisfaction, in turn enhances compassionate attitudes, altruistic behaviours, and higher levels of PM, IM and PSM.^29,44,83,86,87^

 (C) In a context characterised by a short-term orientation culture, where emphasis is placed on immediate outcomes and serving the community, (I) the interaction of (A) the nurses and physicians with the patients may be considered as (M) an act* of patriotism and national pride and* may trigger a commitment *to serve others *and may (O) positively enhance HWs’ IM and PSM (eg, Ghana and Morocco).^29,80^

 (C) In an environment characterised by a short-term orientation culture,^67^ such as Burkina Faso, Uganda and Kenya, serving the community has a flip side. When (A) nurses and physicians are not part of the community they are serving, they are often (I) disregarded (not being part of a family or clan, going against the sacrosanct tradition), which may (M) reduce the self-esteem of HW (poorly accepted) and self-efficacy and mistrust between HW and communities and may (O) contribute to decreased IM and increased intention to migrate or to change jobs (internal migration).^44,82,83^

## Discussion

**Figure 2:**
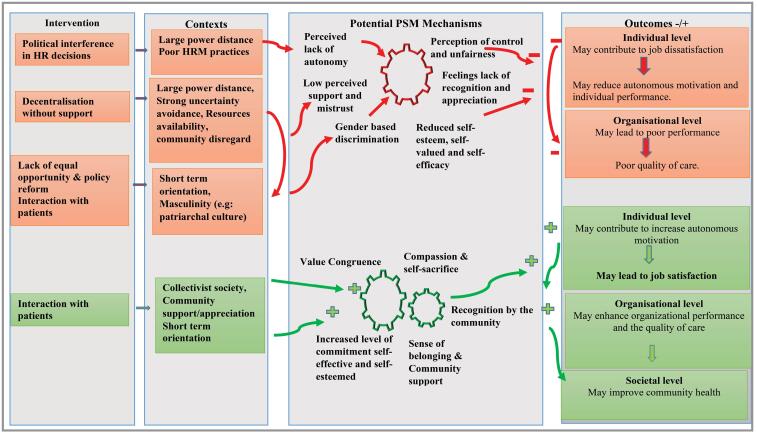
Potential ICAMO Configurations: Societal Culture and Contextual Elements on Health Workers’ Autonomous Motivation (IM, PM, and PSM). Abbreviations: PSM, public service motivation; PM, prosocial motivation; IM, intrinsic motivation; HRM, human resource management; ICAMO, Intervention-Context-Actor-Mechanism-Outcome; HR, human resource.

 Our review aimed to explore the role of the societal culture and contextual elements in shaping the expression of PSM and to identify the underlying processes that relate to the societal culture, contextual elements, and PSM among HW in African healthcare settings. We identified different multilevel factors at the macro-level (human resource for health policies, economic situations), Meso-level (traditional top-down managerial practices), and micro-level (related to interpersonal interaction) that may shape the expression of PSM among HW in African healthcare settings (depicted in Figure 2). Figure 2 illustrates the relationship between societal culture and contextual elements on the left-hand column, and how these variables may shape HWs’ autonomous motivation on the right-hand column.

###  Motivation, Societal Culture, and Managerial Practices

 In African healthcare settings, PSM focuses on altruism, commitment to the public interest, and self-sacrifice. Our included studies often used IM and PSM as sides of the same coin in line with Vandenabeele, who stated that PSM is often used interchangeably with PM and IM (both sides of the same coin).^20^ Some scholars found that motivation was expressed as “an inner drive” without specific mention of specific conceptual definitions of (PSM or IM).^44^ In our review, few scholars referred to Hofstede’s five dimensions of societal culture, drivers of HWs’ motivation: the large power distance, high uncertainty avoidance, collectivism, Short-term orientation, masculinity, and contextual elements.^29,44,50,76,77,80,81,83-88^

###  Macro-Level Contextual Elements: Large Power Distance, Economic Constraints, and Managerial Practices 

 At the macro level, cultural dimensions such as power distance, which we refer here as government policies, along with contextual elements such as economic constraints, and managerial practices play a significantrole in shaping HWs’ autonomy and perceived organisational support.^50,85,86,88^ These findings support the results of other scholars, suggesting that a large power distance between HW and decision-makers at the governmental and district level may decrease HWs’ autonomous motivation by reducing their *perceived work autonomy and increasing perceived lack of organisational support, which leads to reduced motivation and increased job dissatisfaction among HW.*^91,92^ We agree with other scholars that a lack of supportive management practices and leadership styles leads to lower job satisfaction and, in turn, reduced PSM.^93^ When managerial practices and regulations restrict HWs’ autonomy, this enhances their perception of a lack of control over their tasks, and perceived social and procedural injustices, and leads to decreased IM.^85,86^ The findings are consistent with the SDT, which posits that individuals seek to fulfil three basic psychological needs (autonomy, competency, and social relatedness).^94^ Whenever these needs are fulfilled, individuals are more autonomously motivated and therefore may enact more prosocial and altruistic behaviours.^1^ Our findings suggest that organisational environments characterised by high power distance and perceived organisational politics (nepotism and clientelism) may decrease the satisfaction of HWs’ autonomy needs, increase the level of perceived procedural injustice, and reduce the perceived organisational support which may lead to adverse outcomes, such as job dissatisfaction^75^ and negatively affect HWs’ performance and the quality of care.^19,95,96^

 Economic constraints such as low salaries and high living costs lead HW to fulfil their basic needs of living to adopt coping strategies, such as informal payments, to make ends meet.^88^ These contextual factors may contribute to the HWs’ feelings of guilt, reduced self-esteem, stress and job dissatisfaction.^88^ Other scholars, in line with organisational justice theory,^97^ highlighted that low salaries and poor working conditions may contribute to psychological distress, which may harm IM, weaken organisational commitment, increase public sector attrition and foster informal payment practices.^98,99^ These findings are consistent with Hofstede’s cultural dimensions, which state that societies with high uncertainty avoidance tend to experience higher levels of stress and may reduce the perception of self-efficacy of HW.^25,67^ Such context fosters a sense of insecurity and instability, which may contribute to job dissatisfaction and may lower HWs’ performance to provide high-quality care and erode trust from the patients or the community.^100^

###  Meso-Level: Contextual Elements Related to the High Uncertainty Avoidance Dimension and PSM in Managerial Practices

 At the meso level,HW reported low organisational support, reflected in limited training, and bad working environments, which may lead to the *perception of a lack of recognition and a lack of perceived organisational support from managers.*^75,76,86,101^ These factors may harm job satisfaction and undermine IM, contributing further to the desire to quit the public sector in search of stability and security in other countries for work and aggravating the “brain drain” of HW who may perceive that they are not sufficiently valued and recognised.^76,86^ As described by the cultural theory of Hofstede, in a society with high uncertainty avoidance, individuals experience high levels of stress and anxiety due to the fear of the unknown related to the lack of organisational support and ineffective managerial practices.^67^ Findings from a study in Egypt support these findings, showing that alignment between managerial practices and staff values operationalised as person-organisation fit mediates positive outcomes, such as organisational commitment while mitigating adverse outcomes like workplace stress.

###  Organisational Management Practices at Central and District Levels 

 HW refer to inadequate HRM at both central and district levels as a lack of policy reforms and a lack of transparency.^75,85,88^ This resulted in a shortage of human resources, and insufficient safety measures, which increased workload, contributed to poor working environments, and may undermined HWs’ autonomous motivation.

 Our review suggests that poor communication, lack of participatory decision-making, and perceived lack of transparency regarding policy reforms and HRM regulations lead to reinforcing perceptions of exclusion from decision-making processes, reduced *self-esteem, and self-value,* and this may result in undermining HWs’ autonomous motivation.^76,79,84-86,102^

###  Masculinity and Managerial Practices

 Tradition, gender-based discrimination, and spiritual practices are considered as institutional antecedents of variations in PSM expression.^49,67,103,104^ Traditions and spiritual practices tend to discriminate against female HW and may limit their opportunities for career advancement and contribute to unequal distribution of tasks, which may foster *a perception of unfairness*, and *undervaluation*, and may harm their IM.^44,77,83^ The study of Sheikh et al reported that female HW felt that procedural injustice and inequity may lead to decreased autonomous motivation and tensions in the workplace^77^ when the context is characterised by a patriarchal culture and traditional norms often referred to as a “glass ceiling” restricting upward mobility of women into leadership positions.^44,77^ Female HW may feel that their “inputs and outcomes” are less important than those of their male colleagues.^44,77^ This could lead to a bad working environment, such as “tension,” and they may experience dissatisfaction, affecting their autonomous motivation.^44,77^

###  Micro-Level Contextual Elements: Value Congruence, Collectivism, and the Role of Community Appreciation

 At the micro-level, interpersonal interactions and community values play a critical role in shaping HWs’ motivation. Our review has shown that public employees’ identities may be closely connected to the public values of their institution, referred to as *“value congruence*.”^105^ Value congruence explains why HW are intrinsically motivated to act in ways that are consistent with their internal value system and cultural identity, societal norms, and practices that promote serving the community and helping others.^29,51,67,87,106^ Collectivism understood as a sense of belonging to a clan or community and a high degree of social cohesion, may increase the IM of HW.^31,104^

 In some studies, HW underscored the role of *community appreciation* in increasing their IM regardless of the role of financial incentives.^44,79,88^ The feeling of being valued and respected by the community may enhance a sense of belonging and social support and may significantly influence positively job satisfaction and the commitment to perform high-quality care regardless of workplace challenges.^44,79,87,88^ These findings underscore the importance of satisfaction of *“*social *relatedness” needs*, a sense of belonging to a social group integrated into the SDT.^107^ Based on this theory, collectivism may closely relate to individual needs for relatedness as reported in a similar study in Zambia.^107,108^ and public servants in collectivistic societies such as Ghana, Egypt, and Morocco who expressed a strong willingness to commit to serving the public good and the desire to contribute to the community’s well-being.^29,77,81^

 It is argued that HW in collectivistic societies are often intrinsically motivated to serve their communities due to the congruence between personal belief systems and values transmitted throughout education (family) and socialisation processes with PSM motives (civic duty, altruism, self-sacrifice).^29,77,81^ Such socialisation processes are in line with the literature about the antecedents of PSM.^48,109^ From an institutional theory perspective of PSM, national culture shapes certain types of behaviours both directly (through the cultural values prevalent in society) and indirectly through the institutions to which attributes of culture give meanings.^110^

 The expression of PSM is facilitated in collectivistic societies through social identification processes.^33,67,77,111^ In a workplace characterised by supportive human resource for health policies and management and the absence of deviant work unethical practices, value congruence of own belief systems and the work context may increase *the feeling of being self-effective and self-esteemed,* which may enhance the satisfaction of basic psychological needs.^29,44,50,84^

 In line with Brenya et al, national culture may shape the expression of HWs’ PSM if it is congruent with the individual shared belief systems^80^ and when there is the right person-organisation fit.^81^ For instance, in Ghana and Egypt, evidence suggests that cultural expectations, societal norms and community orientation may shape public servants’ PSM and commitment to deliver efficient public service.^80,81^ In contrast, when HW felt undervalued, unsupported, and not recognised by the community related to their gender, tradition,^44,75,86^ and in the context of poor value congruence and inadequate person-organisation fit, this often led to decreased PSM and autonomous motivation, job dissatisfaction, and overreliance on extrinsic incentives.^44,75,84,86,112^

###  Implications for Practice

 Fostering transparency within healthcare systems may reduce the perception of inequity and help build trust, aligning organisational practices with collectivist values and sociocultural contexts.

 To improve and sustain HWs’ PSM in African healthcare settings, policy-makers and managers need to prioritise the transparency, autonomy and participatory HRM that enhance perceived fairness, organisational support, and the fulfilment of the psychological need for autonomy. Highlighting non-monetary incentives such as community appreciation and value congruence may strengthen PSM, particularly in a collectivist culture. These implications, consistent with the application principles of PSM theory, which emphasise the role of embedding public values into institutional practices, may create supportive environments that reinforce HWs’ commitment to public service.^113^

###  Research Implications and Gaps

 In line with Kim,^114^ we suggest that only partial evidence supports the relationship between context, national culture, organisational antecedents and PSM. These connections are complex, interwoven and may necessitate the use of complexity-sensitive research designs, such as realist evaluation,^115^ Qualitative comparative analysis (Charles Ragin) and ethnographic studies, such as in the Uganda study.^116^ We agree with scholars who called for expanding the focus on the underlying mechanisms by which religious values, spiritual practice, and education influence the cultural antecedents and the expression of PSM and how this might be explained by the role of identity formation of HW.^29,117^

 In line with other scholars,^81^ we call for more research on the contribution of PSM and collectivism that may provide more research avenues on how to adapt HRM to the context of a collectivistic society like local culture in North African countries, such as in Egypt or Morocco. More research is still needed to conceptualise what PSM means to HW in African countries.

 Further attention needs to be paid to the role of the socialisation processes of HW to promote prosocial behaviours in a context where the sustainability of financial incentives is not guaranteed.^44,75,82,83^ There is also a need to reduce the value incongruence between managerial practices and the shared values of HW in African healthcare settings. This can be done by using cross-cultural management practices in institutions, which are key to reducing cultural mismatch and creating an environment that fosters collaboration and teamwork, leading to a larger range of employees succeeding in their work and improving gender-based equality.^77,82^ In healthcare settings like Ghana, Somalia, and Burkina-Faso, where gender norms may negatively affect HWs’ autonomous motivation levels, gender-sensitive policies can be addressed by promoting gender equality, fair opportunities for career advancement, and a more inclusive and supportive work environment.^44,76,77^

 This study has some limitations. In addition to the scarcity of PSM research in Africa and the limitations of Hofstede’s framework (static and oversimplification of cultural differences, inconsistencies between his categories, lack of empirical evidence), may have created a tunnel vision around cultural dimensions.^118^ We limited the included studies to those published in French, English, and Arabic, as these are the working languages of the authors, allowing for accurate screening and data extraction in accordance with methodological guidance that recommends aligning language criteria with the authors’ capacity.^65^ However, no eligible studies in French and Arabic met the inclusion criteria during screening. We excluded grey literature and conference proceedings to manage the workload and the feasibility of the study. This exclusion represents a limitation, as this type of literature is sensitive to cultural and societal intangible drivers of motivation.^119^

 Additionally, the selected studies cover mainly East, North, and West African countries, potentially limiting the diversity of socio-cultural contexts represented, as healthcare challenges may differ significantly across African healthcare settings. The findings may not fully capture the diverse socio-cultural context realities shaping HWs’ motivation across the African continent. We provide exploratory relationships. Further systematic review is needed to assess the influence of socio-cultural contexts on HWs’ PSM. According to the concept of analytical generalization, the insights derived from this review are intended to inform theoretical understanding and practical implications that can be applicable to similar contexts, rather than to be extrapolated broadly across all African healthcare settings.^120^

## Conclusion

 This study provides an overview of how PSM is expressed among HW and shaped by healthcare settings within the African context on the basis of a scoping review of the relationship of societal culture, managerial practices and contextual elements. While intrinsic, prosocial and altruistic behaviours are recognised as key drivers of HWs’ commitment to public service delivery, few studies have addressed in depth the links between societal culture, contextual elements, and PSM of HW in African healthcare settings. More research is still needed to unpack the black box of how contextual elements and shared individual beliefs hinder or facilitate the effectiveness of motivational strategies merely focused on performance financing schemes commonly used in LMIC, particularly in Africa. Such schemes may fail to take into consideration societal culture and contextual elements that shape HWs’ motivation. Understanding these black boxes is important for developing culturally congruent and contextually responsive strategies that foster motivation and strengthen public service delivery among HW across diverse African healthcare settings.

## Acknowledgments

 Sincere gratitude to the Mohammed VI Foundation of Sciences and Health, Mohammed VI University of Sciences and Health (UM6SS), Casablanca, Morocco; the Mohammed VI International School of Public Health; and the Mohammed VI Center for Research & Innovation, Rabat, Morocco. This research will contribute towards a Doctor of Philosophy (PhD) degree for Djibrine Diallo at the Doctoral School Center, Laboratory of Public Health and Management Department of Mohammed VI University of Sciences and Health.

## Ethical issues

 Ethical approval for this type of study is not required, as this study is a literature review; we did not interact with the study population.

## Conflicts of interest

 Authors declare that they have no conflicts of interest.

## Data availability statement

 The data contributed to this study are included in the article (Supplementary file 1); further inquiries can be addressed to the corresponding author, Djibrine Diallo, on reasonable request.

## Supplementary files


Supplementary file 1 contains Table S1.

Supplementary file 2 contains Table S2.

Supplementary file 3 contains Figure S1.

Supplementary file 4 contains Table S3.

Supplementary file 5 contains Tables S4 and S5.

Supplementary file 6 contains Box S1.

Supplementary file 7 contains Box S2.

Supplementary file 8 contains Figure S2.

Supplementary file 9 contains Table S6.

## References

[1] Muthuri R, Senkubuge F, Hongoro C (2020). Determinants of motivation among healthcare workers in the East African community between 2009-2019: a systematic review. Healthcare (Basel).

[2] World Health Organization (WHO). 2021 Annual Report of the Alliance for Health Policy and Systems Research. WHO; 2022.

[3] Franco LM, Bennett S, Kanfer R (2002). Health sector reform and public sector health worker motivation: a conceptual framework. Soc Sci Med.

[4] Bertone MP (2018). [Strategies of health workforce retention in rural areas of seven countries of francophone Africa]. Sante Publique.

[5] Ditlopo P, Blaauw D, Rispel LC, Thomas S, Bidwell P (2013). Policy implementation and financial incentives for nurses in South Africa: a case study on the occupation-specific dispensation. Glob Health Action.

[6] Willis-Shattuck M, Bidwell P, Thomas S, Wyness L, Blaauw D, Ditlopo P (2008). Motivation and retention of health workers in developing countries: a systematic review. BMC Health Serv Res.

[7] Buchan J, Aiken L (2008). Solving nursing shortages: a common priority. J Clin Nurs.

[8] Prytherch H, Leshabari MT, Wiskow C (2012). The challenges of developing an instrument to assess health provider motivation at primary care level in rural Burkina Faso, Ghana and Tanzania. Glob Health Action.

[9] Jaskiewicz W, Tulenko K (2012). Increasing community health worker productivity and effectiveness: a review of the influence of the work environment. Hum Resour Health.

[10] Ayalew F, Kibwana S, Shawula S (2019). Understanding job satisfaction and motivation among nurses in public health facilities of Ethiopia: a cross-sectional study. BMC Nurs.

[11] Rowe AK, Rowe SY, Peters DH, Holloway KA, Ross-Degnan D (2021). The effectiveness of training strategies to improve healthcare provider practices in low-income and middle-income countries. BMJ Glob Health.

[12] World Health Organization (WHO). Global Strategy on Human Resources for Health: Workforce 2030. Geneva: WHO; 2016. https://iris.who.int/handle/10665/250368. Accessed April 15, 2025.

[13] Lee MT, Raschke RL (2016). Understanding employee motivation and organizational performance: arguments for a set-theoretic approach. J Innov Knowl.

[14] Paul E, Bodson O, Ridde V (2021). What theories underpin performance-based financing? A scoping review. J Health Organ Manag.

[15] Grant AM (2007). Relational job design and the motivation to make a prosocial difference. Acad Manage Rev.

[16] Bawole JN, Mensah JK, Amegavi GB (2019). Public service motivation scholarship in Africa: a systematic review and research agenda. Int J Public Adm.

[17] Perry JL, Wise LR (1990). The motivational bases of public service. Public Adm Rev.

[18] Ryan RM, Deci EL (2000). Intrinsic and extrinsic motivations: classic definitions and new directions. Contemp Educ Psychol.

[19] Ryan RM, Deci EL (2000). Self-determination theory and the facilitation of intrinsic motivation, social development, and well-being. Am Psychol.

[20] Vandenabeele W, Ritz A, Neumann O. Public service motivation: state of the art and conceptual cleanup. In: Ongaro E, Van Thiel S, eds. The Palgrave Handbook of Public Administration and Management in Europe. London: Palgrave Macmillan; 2018:261-278. doi: 10.1057/978-1-137-55269-3_13.

[21] Lohmann J, Houlfort N, De Allegri M (2016). Crowding out or no crowding out? A self-determination theory approach to health worker motivation in performance-based financing. Soc Sci Med.

[22] Ayalew E, Workineh Y, Abate A, Zeleke B, Semachew A, Woldegiorgies T (2021). Intrinsic motivation factors associated with job satisfaction of nurses in three selected public hospitals in Amhara regional state, 2018. Int J Afr Nurs Sci.

[23] Weldegebriel Z, Ejigu Y, Weldegebreal F, Woldie M (2016). Motivation of health workers and associated factors in public hospitals of West Amhara, Northwest Ethiopia. Patient Prefer Adherence.

[24] Berdud M, Cabasés JM, Nieto J (2016). Incentives and intrinsic motivation in healthcare. Gac Sanit.

[25] Belrhiti Z, Van Damme W, Belalia A, Marchal B (2020). Unravelling the role of leadership in motivation of health workers in a Moroccan public hospital: a realist evaluation. BMJ Open.

[26] Brock JM, Lange A, Leonard KL (2016). Generosity and prosocial behavior in healthcare provision: evidence from the laboratory and field. J Hum Resour.

[27] Munga MA, Maestad O (2009). Measuring inequalities in the distribution of health workers: the case of Tanzania. Hum Resour Health.

[28] Diaconu K, Falconer J, Verbel A, Fretheim A, Witter S (2021). Paying for performance to improve the delivery of health interventions in low- and middle-income countries. Cochrane Database Syst Rev.

[29] Belrhiti Z, Van Damme W, Belalia A, Marchal B (2019). Does public service motivation matter in Moroccan public hospitals? A multiple embedded case study. Int J Equity Health.

[30] Perry JL (1996). Measuring public service motivation: an assessment of construct reliability and validity. J Public Adm Res Theory.

[31] Hondeghem A, Vandenabeele W (2005). Valeurs et motivations dans le service public: perspective comparative. Revue Francaise d’Administration Publique.

[32] Giauque D, Ritz A, Varone F, Anderfuhren-Biget S, Waldner C (2011). La mise en contexte de la motivation à l’égard du service public Comment concilier universalisme et particularisme. Revue Internationale des Sciences Administratives.

[33] Vandenabeele W (2007). Toward a public administration theory of public service motivation. Public Manag Rev.

[34] Fernandes A, Santinha G, Forte T (2022). Public service motivation and determining factors to attract and retain health professionals in the public sector: a systematic review. Behav Sci (Basel).

[35] Hondeghem A, Vandenabeele W (2005). Values and motivation in public administration: public service motivation (PSM) in an international comparative perspective. Rev Fr Administr Publique.

[36] Camilleri E (2007). Antecedents affecting public service motivation. Pers Rev.

[37] van Loon NM, Leisink P, Vandenabeele W (2013). Talking the talk of public service motivation: how public organization logics matter for employees’ expressions of PSM. Int J Public Adm.

[38] Melnik E, Guillemot D (2010). Vers une convergence du management public-privé? Une revue de littérature économique. Revue Française d’Economie.

[39] Schultz PP, Ryan RM. The “why,” “what,” and “how” of healthy self-regulation: mindfulness and well-being from a self-determination theory perspective. In: Ostafin BD, Robinson MD, Meier BP, eds. Handbook of Mindfulness and Self-Regulation. New York: Springer; 2015:81-94. doi: 10.1007/978-1-4939-2263-5_7.

[40] Stefurak T, Morgan R, Johnson RB (2020). The relationship of public service motivation to job satisfaction and job performance of emergency medical services professionals. Public Pers Manag.

[41] Ritz A, Schott C, Nitzl C, Alfes K (2020). Public service motivation and prosocial motivation: two sides of the same coin?. Public Manag Rev.

[42] Mathauer I, Imhoff I (2006). Health worker motivation in Africa: the role of non-financial incentives and human resource management tools. Hum Resour Health.

[43] Honda A, Krucien N, Ryan M (2019). For more than money: willingness of health professionals to stay in remote Senegal. Hum Resour Health.

[44] Prytherch H, Kagoné M, Aninanya GA (2013). Motivation and incentives of rural maternal and neonatal health care providers: a comparison of qualitative findings from Burkina Faso, Ghana and Tanzania. BMC Health Serv Res.

[45] Belle N, Cantarelli P (2014). Monetary Incentives, Motivation, and job effort in the public sector: an experimental study with Italian government executives. Rev Public Pers Adm.

[46] Lohmann J, Muula AS, Houlfort N, De Allegri M (2018). How does performance-based financing affect health workers’ intrinsic motivation? A self-determination theory-based mixed-methods study in Malawi. Soc Sci Med.

[47] Gergen J, Rajkotia Y, Lohmann J, Ravishankar N (2018). Performance-based financing kick-starts motivational “feedback loop”: findings from a process evaluation in Mozambique. Hum Resour Health.

[48] Wang TM, van Witteloostuijn A, Heine F (2020). A moral theory of public service motivation. Front Psychol.

[49] Perry JL, Brudney JL, Coursey D, Littlepage L (2008). What drives morally committed citizens? A study of the antecedents of public service motivation. Public Adm Rev.

[50] Belrhiti Z, Van Damme W, Belalia A, Marchal B (2020). The effect of leadership on public service motivation: a multiple embedded case study in Morocco. BMJ Open.

[51] Mussagulova A, van der Wal Z (2021). “All still quiet on the non-Western front?” Non-Western public service motivation scholarship: 2015-2020. Asia Pac J Public Adm.

[52] van der Wal Z (2015). “All quiet on the non-Western front?” A review of public service motivation scholarship in non-Western contexts. Asia Pac J Public Adm.

[53] Neumann O, Ritz A (2015). Public service motivation and rational choice modelling. Public Money Manag.

[54] Leonard KL, Masatu MC (2010). Using the Hawthorne effect to examine the gap between a doctor’s best possible practice and actual performance. J Dev Econ.

[55] Perry JL, Hondeghem A, Wise LR (2010). Revisiting the motivational bases of public service: twenty years of research and an agenda for the future. Public Adm Rev.

[56] Temba RS (2025). The factors influencing public service motivation among public servants in Tanzania Ministry of Health. Int J Sci Res Arch.

[57] Robert G, Fulop N. The Role of Context in Successful Improvement. London: The Health Foundation; 2014.

[58] Marchal B, van Belle S, van Olmen J, Hoerée T, Kegels G (2012). Is realist evaluation keeping its promise? A review of published empirical studies in the field of health systems research. Evaluation.

[59] Ritz A, Brewer GA (2013). Does societal culture affect public service motivation? Evidence of sub-national differences in Switzerland. Int Public Manag J.

[60] Levac D, Colquhoun H, O’Brien KK (2010). Scoping studies: advancing the methodology. Implement Sci.

[61] Arksey H, O’Malley L (2005). Scoping studies: towards a methodological framework. Int J Soc Res Methodol.

[62] World Bank Data Help Desk. World Bank Country and Lending Groups. https://datahelpdesk.worldbank.org/knowledgebase/articles/906519-world-bank-country-and-lending-groups. Accessed March 8, 2023.

[63] Peters MD, Godfrey CM, Khalil H, McInerney P, Parker D, Soares CB (2015). Guidance for conducting systematic scoping reviews. Int J Evid Based Healthc.

[64] Cohen J (1960). A coefficient of agreement for nominal scales. Educ Psychol Meas.

[65] JBI Global Wiki. JBI Reviewer’s Manual. 2020. https://jbi-global-wiki.refined.site/download/attachments/355863557/JBI_Reviewers_Manual_2020June.pdf?download=true. Accessed May 16, 2025.

[66] Tricco AC, Lillie E, Zarin W (2018). PRISMA Extension for Scoping Reviews (PRISMA-ScR): Checklist and Explanation. Ann Intern Med.

[67] Hofstede G (2011). Dimensionalizing cultures: the Hofstede model in context. Online Readings in Psychology and Culture.

[68] Barnett-Page E, Thomas J (2009). Methods for the synthesis of qualitative research: a critical review. BMC Med Res Methodol.

[69] Rees CE, Proctor DW, Nguyen VN, Ottrey E, Mattick KL (2025). Realist analysis of qualitative data in health professions education research. Med Educ.

[70] Dopfer K, Foster J, Potts J (2004). Micro-meso-macro. J Evol Econ.

[71] Nowell LS, Norris JM, White DE, Moules NJ (2017). Thematic analysis: striving to meet the trustworthiness criteria. Int J Qual Methods.

[72] Braun V, Clarke V (2006). Using thematic analysis in psychology. Qual Res Psychol.

[73] Astbury B, Leeuw FL (2010). Unpacking black boxes: mechanisms and theory building in evaluation. Am J Eval.

[74] Marchal B, Kegels G, van Belle S. Theory and realist methods. In: Emmel N, Greenhalgh J, Manzano A, Monaghan M, Dalkin S, eds. Theory and Realist Methods. SAGE Publications Ltd. 2018:79-90. doi: 10.4135/9781526451729.n6.

[75] Hopwood H, Harris D, Sevalie S, Iyawa G, Langan Martin J (2023). The barriers and facilitators to decentralised nurse-led mental health service delivery in Sierra Leone. Community Ment Health J.

[76] Adjei-Mensah S (2023). Factors influencing brain drain among health workers in Ghana. Eur J Hum Resour.

[77] Sheikh NS, Gele A (2023). Factors influencing the motivation of maternal health workers in conflict setting of Mogadishu, Somalia. PLOS Glob Public Health.

[78] Saisai R, Boufalta MS (2022). The impact of the COVID-19 pandemic on nurses’ motivation at work: a field study at the public hospital in Ali Mendjeli, Algeria Journal of Financial,. Accounting and Managerial Studies.

[79] Witter S, Wurie H, Namakula J, Mashange W, Chirwa Y, Alonso-Garbayo A (2018). Why do people become health workers? Analysis from life histories in 4 post-conflict and post-crisis countries. Int J Health Plann Manage.

[80] Brenya E, Adu-Gyamfi S, Kyei EY (2017). Public service motivation in Ghanaian civil service: a case study of Ejisu-Juabeng municipality. Int J Public Adm.

[81] Gould-Williams JS, Sayed Mostafa AM, Bottomley P (2015). Public service motivation and employee outcomes in the Egyptian public sector: testing the mediating effect of person-organization fit. J Public Adm Res Theory.

[82] Okuga M, Kemigisa M, Namutamba S, Namazzi G, Waiswa P (2015). Engaging community health workers in maternal and newborn care in eastern Uganda. Glob Health Action.

[83] Ojakaa D, Olango S, Jarvis J (2014). Factors affecting motivation and retention of primary health care workers in three disparate regions in Kenya. Hum Resour Health.

[84] Prytherch H, Kakoko DC, Leshabari MT, Sauerborn R, Marx M (2012). Maternal and newborn healthcare providers in rural Tanzania: in-depth interviews exploring influences on motivation, performance and job satisfaction. Rural Remote Health.

[85] Songstad NG, Rekdal OB, Massay DA, Blystad A (2011). Perceived unfairness in working conditions: the case of public health services in Tanzania. BMC Health Serv Res.

[86] Luboga S, Hagopian A, Ndiku J, Bancroft E, McQuide P (2011). Satisfaction, motivation, and intent to stay among Ugandan physicians: a survey from 18 national hospitals. Int J Health Plann Manage.

[87] Serneels P, Montalvo JG, Pettersson G, Lievens T, Butera JD, Kidanu A (2010). Who wants to work in a rural health post? The role of intrinsic motivation, rural background and faith-based institutions in Ethiopia and Rwanda. Bull World Health Organ.

[88] Stringhini S, Thomas S, Bidwell P, Mtui T, Mwisongo A (2009). Understanding informal payments in health care: motivation of health workers in Tanzania. Hum Resour Health.

[89] Sayed Mostafa AM, Gould-Williams JS, Bottomley P (2015). High-performance human resource practices and employee outcomes: the mediating role of public service motivation. Public Adm Rev.

[90] Hofstede G. Culture’s Consequences: International Differences in Work-Related Values. SAGE Publications; 1984.

[91] Dieleman M, Gerretsen B, van der Wilt GJ (2009). Human resource management interventions to improve health workers’ performance in low- and middle-income countries: a realist review. Health Res Policy Syst.

[92] Mbindyo P, Gilson L, Blaauw D, English M (2009). Contextual influences on health worker motivation in district hospitals in Kenya. Implement Sci.

[93] Ng K, Franken E, Nguyen D, Teo S (2023). Job satisfaction and public service motivation in Australian nurses: the effects of abusive supervision and workplace bullying. Int J Hum Resourc Manag.

[94] Vandenabeele W (2014). Explaining public service motivation: the role of leadership and basic needs satisfaction. Rev Public Pers Adm.

[95] Eisenberger R, Fasolo P, Davis-LaMastro V (1990). Perceived organizational support and employee diligence, commitment, and innovation. J Appl Psychol.

[96] Gagné M, Deci EL (2005). Self-determination theory and work motivation. J Organ Behav.

[97] Greenberg J (1990). Organizational justice: yesterday, today, and tomorrow. J Manage.

[98] Aberese-Ako M, van Dijk H, Gerrits T, Arhinful DK, Agyepong IA (2014). ‘Your health our concern, our health whose concern?’: perceptions of injustice in organizational relationships and processes and frontline health worker motivation in Ghana. Health Policy Plan.

[99] Mutale W, Ayles H, Bond V, Mwanamwenge MT, Balabanova D (2013). Measuring health workers’ motivation in rural health facilities: baseline results from three study districts in Zambia. Hum Resour Health.

[100] Rojo J, Everett B, Ramjan LM, Hunt L, Salamonson Y (2020). Hofstede’s cultural dimensions as the explanatory framework for performance issues during clinical placement: a mixed methods study. Nurse Educ Today.

[101] Emerson DJ. Organizational Culture, Job Satisfaction and Turnover Intentions: The Mediating Role of Perceived Organizational Support [dissertation]. Virginia Commonwealth University; 2013.

[102] Ogunleye AJ, Osekita DA (2016). Effect of job status, gender, and employees’ achievement motivation behavior on work performance: a case study of selected local government employees in Ekiti state, Nigeria. Eur Sci J.

[103] Baird KM, Tung A, Yu Y (2019). Employee organizational commitment and hospital performance. Health Care Manage Rev.

[104] Najera M (2008). Managing Mexican workers: implications of Hofstede’s cultural dimensions. J Int Bus Res.

[105] Giauque D, Anderfuhren-Biget S, Varone F (2015). HRM practices sustaining PSM: when values congruency matters. Int J Public Sect Perform Manag.

[106] Johnson KA, Hook JN, Davis DE, Van Tongeren DR, Sandage SJ, Crabtree SA (2016). Moral foundation priorities reflect US Christians’ individual differences in religiosity. Pers Individ Dif.

[107] Ryan RM, Deci EL. Self-Determination Theory: Basic Psychological Needs in Motivation, Development, and Wellness. Guilford Publications; 2017.

[108] Manda K, Silumbwe A, Mupeta Kombe M, Hangoma P (2023). Motivation and retention of primary healthcare workers in rural health facilities: an exploratory qualitative study of Chipata and Chadiza districts, Zambia. Glob Public Health.

[109] Perry JL (1997). Antecedents of public service motivation. J Public Adm Res Theory.

[110] Kreiser PM, Marino LD, Dickson P, Weaver KM (2010). Cultural influences on entrepreneurial orientation: the impact of national culture on risk taking and proactiveness in SMEs. Entrep Theory Pract.

[111] Jing L (2022). A cross-cultural study on the film the treatment from the perspective of Geert Hofstede’s cultural dimensions. BCP Soc Sci Humanit.

[112] Muthuri R, Senkubuge F, Hongoro C (2020). An investigation of healthcare professionals’ motivation in public and mission hospitals in Meru county, Kenya. Healthcare (Basel).

[113] Paarlberg L, Perry JL, Hondeghem A. From theory to practice: Strategies for applying public service motivation. In: Perry JL, Hondeghem A, eds. Motivation in Public Management: The Call of Public Service. Oxford University Press. 2008:268-293. doi: 10.1093/oso/9780199234035.003.0014.

[114] Kim S (2017). National culture and public service motivation: investigating the relationship using Hofstede’s five cultural dimensions. Int Rev Adm Sci.

[115] Tilley N, Pawson R. Realistic Evaluation. SAGE Publications. 1997:1-256.

[116] Rowe AK, de Savigny D, Lanata CF, Victora CG (2005). How can we achieve and maintain high-quality performance of health workers in low-resource settings?. Lancet.

[117] Dewi DS, Nurmandi A, Mutiarin D, Jovita HD, Salahudin S, Djuwitaningsih EW (2020). Bringing Religious Value to Public Service Motivation. Int J Innov Creat Chang.

[118] Signorini P, Wiesemes R, Murphy R (2009). Developing alternative frameworks for exploring intercultural learning: a critique of Hofstede’s cultural difference model. Teach High Educ.

[119] Chandna K, Vine MM, Snelling SJ, Harris R, Smylie J, Manson H (2019). Principles, approaches, and methods for evaluation in Indigenous contexts: a grey literature scoping review. Can J Program Eval.

[120] Yin RK. Case Study Research and Applications: Design and Methods. SAGE Publications; 2017.

