# Supplementary material for: How Organisational and Socio-Cultural Contexts Shape Healthcare Workers’ Intrinsic, Prosocial, and Public Service Motivation in Africa: A Scoping Review
Source: Int J Health Policy Manag. 2025 Oct 4;14:8861. doi: 10.34172/ijhpm.8861 (PMC12958157; doi:10.34172/ijhpm.8861)
Supplement: Supplementary file 1 — contains Table S1. [file ijhpm-14-8861-s001.pdf]

Article title: How Organisational and Socio-Cultural Contexts Shape Healthcare Workers' Intrinsic, Prosocial, and Public Service Motivation in Africa: A Scoping Review

Journal name: International Journal of Health Policy and Management (IJHPM)

Authors' information: Djibrine Diallo[1,2]*, Bruno Marchal[3], Zakaria Belrhiti[1,2]

[1]Mohammed VI International School of Public Health, Mohammed VI University of Sciences and Health, Casablanca, Morocco.

[2]Laboratory of Public Health and Management Department, Mohammed VI Center for Research & Innovation, Rabat, Morocco.

[3]Department of Public Health, Institute of Tropical Medicine, Antwerp, Belgium.

***Correspondence to:** Djibrine Diallo; Email: ddiallo@um6ss.ma

Citation: Diallo D, Marchal B, Belrhiti Z. How organisational and socio-cultural contexts shape healthcare workers' intrinsic, prosocial, and public service motivation in Africa: a scoping review. Int J Health Policy Manag. 2025;14:8861. doi:10.34172/ijhpm.8861

Supplementary file 1

Data sources	Search strategy	Date
WEB OF science	Public service motivation or prosocial motivation or intrinsic motivation AND culture and theory and concept and Framework.	04/05/2022
PubMed	Public service motivation OR MSP OR prosocial motivation OR intrinsic motivation AND culture.	04/05/2022
Scopus	Public service motivation OR MSP OR prosocial motivation OR intrinsic motivation AND culture.	04/05/2022
Google scholar	("public service motivation" OR "MSP" OR "prosocial motivation" OR "intrinsic motivation") AND ("culture" OR "collectivism" OR "individualism" OR "uncertainty avoidance" OR "power distance") AND ("health worker " OR "hospital") AND ("Africa")	04/07/2022
	**Updated (Filter from 2022 to 2024)**	01/02/2024
Web of Science	(Public service motivation or prosocial motivation or intrinsic motivation) AND (culture OR collectivism OR individualism OR uncertainty avoidance OR power distance OR masculinity OR femininity OR long-term orientation OR short-term orientation OR indulgence OR restraint) AND (health worker OR healthcare workers OR health professional)	
PubMed	((("Motivation"[Mesh]) AND "Culture"[Mesh]) AND "Health Personnel"[Mesh]) AND ("Africa"[Mesh] OR "Africa, Northern"[Mesh])	01/02/2024
Scopus	(public AND service AND motivation OR msp OR prosocial AND motivation OR intrinsic AND motivation AND culture) filter limited to African countries and undefined studies,	01/02/2024
Google scholar	("public service motivation" OR "prosocial motivation" OR "intrinsic motivation") AND ("culture" OR "collectivism" OR "individualism" OR "uncertainty avoidance" OR "power distance" OR "masculinity" OR "femininity" OR "long-term orientation" OR "short-term orientation" OR "indulgence" OR "restraint") AND ("health worker" OR "healthcare workers "OR "health professional") AND ("africa" OR "african countries" OR "low and middle income countries") filter from 2022 to 2024	01/02/2024
