# Supplementary material for: How Organisational and Socio-Cultural Contexts Shape Healthcare Workers’ Intrinsic, Prosocial, and Public Service Motivation in Africa: A Scoping Review
Source: Int J Health Policy Manag. 2025 Oct 4;14:8861. doi: 10.34172/ijhpm.8861 (PMC12958157; doi:10.34172/ijhpm.8861)
Supplement: Supplementary file 2 — contains Table S2. [file ijhpm-14-8861-s002.pdf]

Article title: How Organisational and Socio-Cultural Contexts Shape Healthcare Workers' Intrinsic, Prosocial, and Public Service Motivation in Africa: A Scoping Review

Journal name: International Journal of Health Policy and Management (IJHPM)

Authors' information: Djibrine Diallo[1,2]*, Bruno Marchal[3], Zakaria Belrhiti[1,2]

[1]Mohammed VI International School of Public Health, Mohammed VI University of Sciences and Health, Casablanca, Morocco.

[2]Laboratory of Public Health and Management Department, Mohammed VI Center for Research & Innovation, Rabat, Morocco.

[3]Department of Public Health, Institute of Tropical Medicine, Antwerp, Belgium.

***Correspondence to:** Djibrine Diallo; Email: ddiallo@um6ss.ma

Citation: Diallo D, Marchal B, Belrhiti Z. How organisational and socio-cultural contexts shape healthcare workers' intrinsic, prosocial, and public service motivation in Africa: a scoping review. Int J Health Policy Manag. 2025;14:8861. doi:10.34172/ijhpm.8861

Supplementary file 2

	**Reviewer 2 Reject**	**Reviewer 2 Accept**	**Total**			
**Reviewer 1 Reject**	1391	7	**1398**			
**Reviewer 1 Accept**	11	37	**48**			
**Title Total**	**1402**	**44**	**1468**			
**Interpretation:**						
**Value of *K***		**Strength of agreement**				
< 0.20		Poor				
0.21 - 0.40		Fair				
0.41 - 0.60		Moderate				
0.61 - 0.80		Good				
0.81 - 1.00		Very good				
**(*P0*) Observed Agreement**	**(*PE*) Expected Agreement**	***K***	***se(K)***	**Lower 95% CI**		**Upper 95% CI**
0,973	0,910	**0,696**	0,047	**0,603**	**to**	**0,789**
