# Supplementary material for: How Organisational and Socio-Cultural Contexts Shape Healthcare Workers’ Intrinsic, Prosocial, and Public Service Motivation in Africa: A Scoping Review
Source: Int J Health Policy Manag. 2025 Oct 4;14:8861. doi: 10.34172/ijhpm.8861 (PMC12958157; doi:10.34172/ijhpm.8861)
Supplement: Supplementary file 3 — contains Figure S1. [file ijhpm-14-8861-s003.pdf]

Article title: How Organisational and Socio-Cultural Contexts Shape Healthcare Workers' Intrinsic, Prosocial, and Public Service Motivation in Africa: A Scoping Review

Journal name: International Journal of Health Policy and Management (IJHPM)

Authors' information: Djibrine Diallo[1,2]*, Bruno Marchal[3], Zakaria Belrhiti[1,2]

[1]Mohammed VI International School of Public Health, Mohammed VI University of Sciences and Health, Casablanca, Morocco.

[2]Laboratory of Public Health and Management Department, Mohammed VI Center for Research & Innovation, Rabat, Morocco.

[3]Department of Public Health, Institute of Tropical Medicine, Antwerp, Belgium.

***Correspondence to:** Djibrine Diallo; Email: ddiallo@um6ss.ma

Citation: Diallo D, Marchal B, Belrhiti Z. How organisational and socio-cultural contexts shape healthcare workers' intrinsic, prosocial, and public service motivation in Africa: a scoping review. Int J Health Policy Manag. 2025;14:8861. doi:10.34172/ijhpm.8861

Supplementary file 3

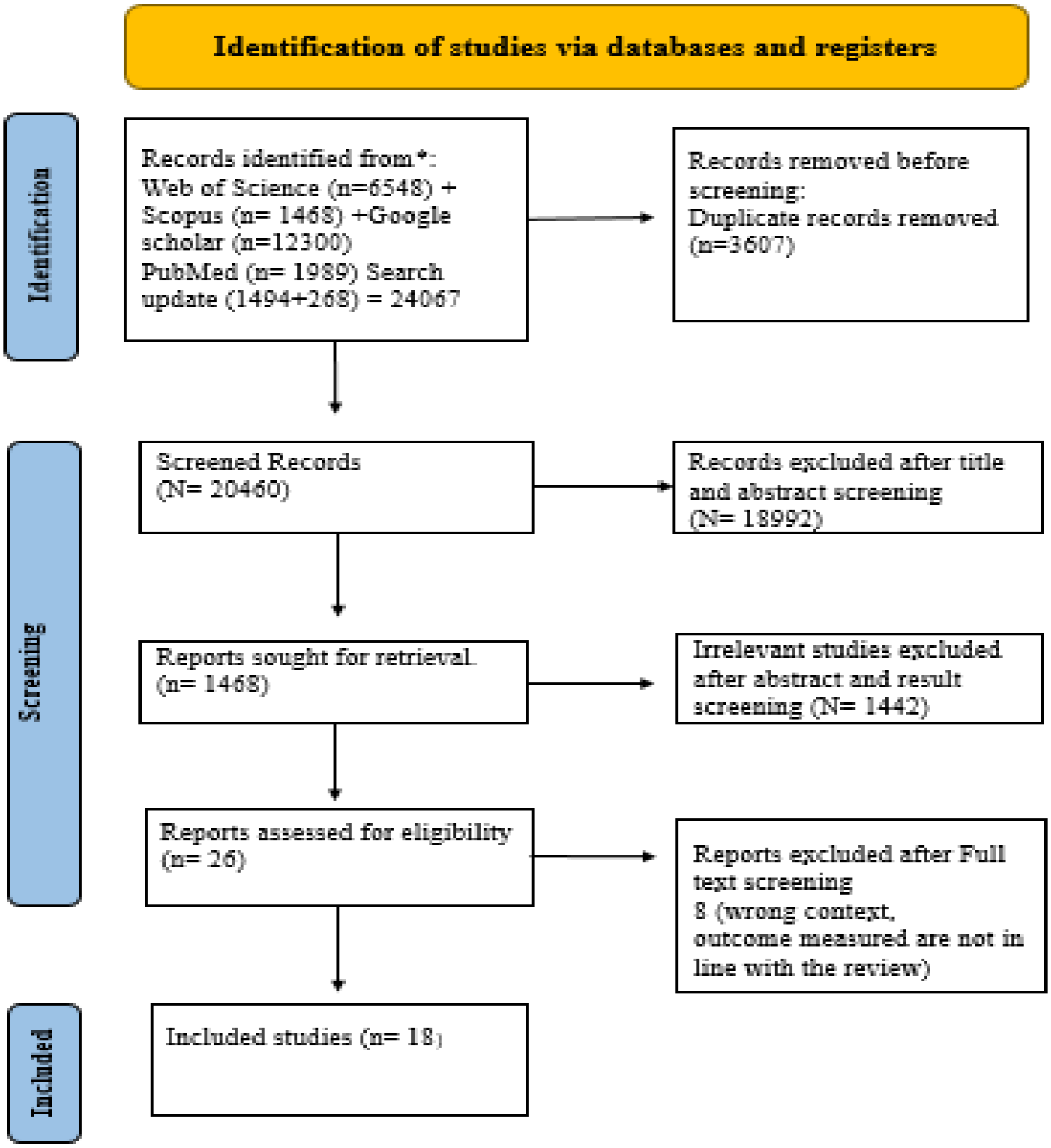
Identification of studies via databases and registers
Identification
Records identified from*: Web of Science (n=6548) + Scopus (n= 1468) +Google scholar (n=12300) PubMed (n= 1989) Search update (1494+268) = 24067
Records removed before screening: Duplicate records removed (n=3607)
Screening
Screened Records (N= 20460)
Records excluded after title and abstract screening (N= 18992)
Reports sought for retrieval. (n= 1468)
Irrelevant studies excluded after abstract and result screening (N= 1442)
Reports assessed for eligibility (n= 26)
Reports excluded after Full text screening 8 (wrong context, outcome measured are not in line with the review)
Included
Included studies (n= 18)
