# Supplementary material for: How Organisational and Socio-Cultural Contexts Shape Healthcare Workers’ Intrinsic, Prosocial, and Public Service Motivation in Africa: A Scoping Review
Source: Int J Health Policy Manag. 2025 Oct 4;14:8861. doi: 10.34172/ijhpm.8861 (PMC12958157; doi:10.34172/ijhpm.8861)
Supplement: Supplementary file 5 — contains Tables S4 and S5. [file ijhpm-14-8861-s005.pdf]

Article title: How Organisational and Socio-Cultural Contexts Shape Healthcare Workers' Intrinsic, Prosocial, and Public Service Motivation in Africa: A Scoping Review

Journal name: International Journal of Health Policy and Management (IJHPM)

Authors' information: Djibrine Diallo[1,2]*, Bruno Marchal[3], Zakaria Belrhiti[1,2]

[1]Mohammed VI International School of Public Health, Mohammed VI University of Sciences and Health, Casablanca, Morocco.

[2]Laboratory of Public Health and Management Department, Mohammed VI Center for Research & Innovation, Rabat, Morocco.

[3]Department of Public Health, Institute of Tropical Medicine, Antwerp, Belgium.

***Correspondence to:** Djibrine Diallo; Email: ddiallo@um6ss.ma

Citation: Diallo D, Marchal B, Belrhiti Z. How organisational and socio-cultural contexts shape healthcare workers' intrinsic, prosocial, and public service motivation in Africa: a scoping review. Int J Health Policy Manag. 2025;14:8861. doi:10.34172/ijhpm.8861

Supplementary file 5

Table S5: definitions of critical Societal cultural dimensions according to Hofstede [67]

**Power Distance**		**Uncertainty Avoidance**		**Individualism versus Collectivism**	
**Small Power Distance**: The source of power requires legitimacy. Older people are necessarily respected or feared. Subordinates expect to be consulted in decisions. Corruption is considered rare. Scandals often end political careers.	**Large Power Distance:** In this culture, the legitimacy of power need not be legitimised. Power sources are co-optation and are changed by revolutions. Subordinates are expected to be told what to do. Corruption is frequent, and scandals are often covered up.	**Low Uncertainty Avoidance:** societies often accept uncertainty, ambiguity, deviant ideas, and new life patterns. They are described as having lower stress and anxiety, higher self-control, and perceived well-being. In such a culture, citizens are considered competent towards the public authorities.	**High Uncertainty Avoidance**: Societies are perceived as reluctant to ambiguity, uncertainty, deviant ideas and people. They emphasise control and certainty over complexity and ambiguity. They abide by strict behavioural codes and often experience higher stress, anxiety, and low perceived well-being. Citizens are considered incompetent by public authorities.	**Individualism**: Individuals are centred on their personal needs and their close family members. Shared values are the "I" – consciousness, right to privacy, and speaking one's mind is healthy.	**Collectivism:** Social cohesion clan-like culture are key characteristics of collectivism, which characterised by values such We "We" –consciousness, social harmony, and loyalty.

Masculinity versus Feminity		Long-Term vs. Short-Term Orientation		Indulgence versus Restrained	
**Feminity**: Men and women should be modest, caring, balanced between family and work, and sympathy for the weak. Both fathers and mothers deal with facts and feelings. Both boys and girls may cry, but neither should they fight. Mothers decide on the number of children.	**Masculinity:** Men should be assertive and ambitious. Work prevails over family. Admiration for the strong, fathers deal with facts, mothers with feelings. Girls cry, boys don't; boys should fight back, girls shouldn't fight. Fathers decide on family size. Few women in elected political positions.	**Short-Term Orientation:** It refers to the importance of fulfilling immediate needs and traditions, which are sacrosanct. A lower tolerance for uncertainty and a higher preference for stability and continuity. The dominant values are being proud of one's country, reciprocating social obligations and service to others, and maintaining personal stability.	**Long-Term Orientation**: reflects society's focus on future-oriented thinking, planning, and perseverance. It is associated with values of traditions; norms are adaptable to changed circumstances.	**Indulgence:** It is due to the importance of values such as freedom and self-expression Indulgent societies tend to have a more relaxed attitude towards social norms.	**Restrained:** it means that societies regulate individual behaviour through social norms, prioritising self-discipline, and tradition and values, conformity to societal expectations.
