# Supplementary material for: How Organisational and Socio-Cultural Contexts Shape Healthcare Workers’ Intrinsic, Prosocial, and Public Service Motivation in Africa: A Scoping Review
Source: Int J Health Policy Manag. 2025 Oct 4;14:8861. doi: 10.34172/ijhpm.8861 (PMC12958157; doi:10.34172/ijhpm.8861)
Supplement: Supplementary file 6 — contains Box S1. [file ijhpm-14-8861-s006.pdf]

Article title: How Organisational and Socio-Cultural Contexts Shape Healthcare Workers' Intrinsic, Prosocial, and Public Service Motivation in Africa: A Scoping Review

Journal name: International Journal of Health Policy and Management (IJHPM)

Authors' information: Djibrine Diallo[1,2]*, Bruno Marchal[3], Zakaria Belrhiti[1,2]

[1]Mohammed VI International School of Public Health, Mohammed VI University of Sciences and Health, Casablanca, Morocco.

[2]Laboratory of Public Health and Management Department, Mohammed VI Center for Research & Innovation, Rabat, Morocco.

[3]Department of Public Health, Institute of Tropical Medicine, Antwerp, Belgium.

***Correspondence to:** Djibrine Diallo; Email: ddiallo@um6ss.ma

Citation: Diallo D, Marchal B, Belrhiti Z. How organisational and socio-cultural contexts shape healthcare workers' intrinsic, prosocial, and public service motivation in Africa: a scoping review. Int J Health Policy Manag. 2025;14:8861. doi:10.34172/ijhpm.8861

Supplementary file 6

- Community appreciation<br>- Contextual element<br>- Importance of the participation in "decision-making"<br>- Lack of autonomy<br>- Lack of policies supporting mental health service<br>- Lack of recognition<br>- Lack of resources<br>- Lack of support and supervision<br>- Lack of training<br>- Low priority of the mental health service<br><br>- Poor salary<br>- Inequalities in allowance distribution as a demotivating factor<br>- Promotional delays as a demotivating factor<br>- Workloads<br>- cultural dimension<br>- Masculinity versus Feminity<br>- Power distance and influence<br>- Uncertainty avoidance	- Gender norm<br>- Importance of good working condition on job satisfaction<br>- Importance of intrinsic motivation "inner motivation"<br>- Family education and religious beliefs<br><br>- Intrinsic motivation<br>- Job satisfaction<br>- Knowledge gap<br>- Mechanism<br>- continuous professional development opportunities<br>- Passion as a driven force for intrinsic motivation<br>- Policies supporting<br>- Outcomes<br>- Passion as a driven force for intrinsic motivation<br>- Poor Working environment and condition<br><br>- Prosocial motivation<br>- Public service motivation Social culture's influence on CHW advice
