# Supplementary material for: How Organisational and Socio-Cultural Contexts Shape Healthcare Workers’ Intrinsic, Prosocial, and Public Service Motivation in Africa: A Scoping Review
Source: Int J Health Policy Manag. 2025 Oct 4;14:8861. doi: 10.34172/ijhpm.8861 (PMC12958157; doi:10.34172/ijhpm.8861)
Supplement: Supplementary file 7 — contains Box S2. [file ijhpm-14-8861-s007.pdf]

Article title: How Organisational and Socio-Cultural Contexts Shape Healthcare Workers' Intrinsic, Prosocial, and Public Service Motivation in Africa: A Scoping Review

Journal name: International Journal of Health Policy and Management (IJHPM)

Authors' information: Djibrine Diallo[1,2]*, Bruno Marchal[3], Zakaria Belrhiti[1,2]

[1]Mohammed VI International School of Public Health, Mohammed VI University of Sciences and Health, Casablanca, Morocco.

[2]Laboratory of Public Health and Management Department, Mohammed VI Center for Research & Innovation, Rabat, Morocco.

[3]Department of Public Health, Institute of Tropical Medicine, Antwerp, Belgium.

***Correspondence to:** Djibrine Diallo; Email: ddiallo@um6ss.ma

Citation: Diallo D, Marchal B, Belrhiti Z. How organisational and socio-cultural contexts shape healthcare workers' intrinsic, prosocial, and public service motivation in Africa: a scoping review. Int J Health Policy Manag. 2025;14:8861. doi:10.34172/ijhpm.8861

Supplementary file 7

Title: okuga2015 (1).pdf
Doc Creator: djibrine.diallo
Doc Date: 6/2/2024
Codes Applied: Social culture's influence on CHW advice
Excerpt Creator: djibrine.diallo
Excerpt Created On: 6/7/2024
Excerpt Range: Page 7: 1495-1648
CHWsand health workers reported that practices were morelikely to be sustained if supported culturally, regardless ofwhat healthcare staff were promoting.
Title: okuga2015 (1).pdf
Doc Creator: djibrine.diallo
Doc Date: 6/2/2024
Codes Applied: Intrinsic motivation
Excerpt Creator: djibrine.diallo
Excerpt Created On: 6/7/2024
Excerpt Range: Page 9: 4716-4987
While CHWs noted that financial incentiveswerekey tojob performance, intrinsic motivation and job satisfactionwere also driving forces. They considered appreciationfrom community members and the supervision theyreceived from formal health workers as important moti-vators.
Title: luboga2010 (2).pdf
Doc Creator: djibrine.diallo
Doc Date: 6/2/2024
Codes Applied: Lack of resources
Excerpt Creator: djibrine.diallo
Excerpt Created On: 6/8/2024
Excerpt Range: Page 9: 835-1362
Then another thing is equipment. We are doing operations but we don't havesome equipment like theatre lights. After complaining we were given a tube foroperation, butevenin thewholewardwe don'thave enough lights. Andcanyouimagine thewhole of this hospital with only two oxygen concentrators? At leastevery ward should be having one or two. We have only one for the pediatric wardaftercomplainingfor solong.Soif youareusingitonthe child,andsomeone elseneeds it you either remove the child to die or you wait for the other to die.

Title: luboga2010 (2).pdf
Doc Creator: djibrine.diallo
Doc Date: 6/2/2024
Codes Applied: Importance of good working condition on job satisfaction
Excerpt Creator: djibrine.diallo
Excerpt Created On: 6/8/2024
Excerpt Range: Page 10: 1179-1885
Actually people are not looking for money when they go away (migrate), theyare looking for good working environment. It is not only the money. Take anexample, people are working in mission hospitals, when you want to do asurgery, things to be done are there, when you have done your good surgery thenurses will follow up the patients very well and you become satis□ed that thepatientshaverecovered,youhavecometoadiagnosiswiththeallthingsthatarerequired and you treat the patients and they recover very well. And those peopleare therenotbecausetheyaregivena lotof money—the health staffsinmissionhospitals are given half the pay of the nurse in public units—but they are therebecause the environment is good.
Title: Witter 2018.pdf
Doc Creator: djibrine.diallo
Doc Date: 6/2/2024
Excerpt Creator: djibrine.diallo
Excerpt Created On: 6/6/2024
Excerpt Range: Page 9: 305-541
These findings reinforce the conclusion thatrecruitment strategies should focus on staff with strong intrinsic motivation, especially to improve retention in hard toserve areas and over periods requiring resilience in the health system.
Title: Witter 2018.pdf
Doc Creator: djibrine.diallo
Doc Date: 6/2/2024
Excerpt Creator: djibrine.diallo
Excerpt Created On: 6/6/2024
Excerpt Range: Page 9: 2531-3035
Gendered expectations also emerged as a theme—both in terms of encouraging entry to “caring” professions andalso in relationto staying in servicein localareas.Thehealth workforce and itsroles arehighly genderedin mostcoun-tries,includingthese13and healthworkforces will continue to be feminised,especiallyatlower levels.Ensuringgenderequity for staff and gender responsive services for users will involve fostering dialogue and action to support changewithin institutions, households, and at policy level.
Title: belrhiti2019 (3).pdf
Doc Creator: djibrine.diallo
Doc Date: 6/2/2024
Codes Applied: Family education and religious beliefs
Excerpt Creator: djibrine.diallo
Excerpt Created On: 6/5/2024
Excerpt Range: Page 9: 964-1735
We found that for many health workers we interviewed,PSM and religious beliefs and values are intertwined.They express their altruistic and compassionate attitudetowards patients as a spiritual personal obligation. Whenserving patients ethically and altruistically, they expectto receive intangible and divine rewards (in arabic hassa-nat’ or ajre). They also frame this as a commitment topublic values such as equity and patriotism.“In Arabicterms, we do this because we need to make our salary“Halal.” [Halal means compliant with Islamic rules].“Thank God.” We said this because it is veryimportant! We must make sure that our salary ishalal before God and our own conscience. There isnobody watching over us, whether we came at 8 amin the morning or not”. EJMH 9 doctor
Title: belrhiti2019 (3).pdf
Doc Creator: djibrine.diallo
Doc Date: 6/2/2024
Excerpt Creator: djibrine.diallo
Excerpt Created On: 6/5/2024
Excerpt Range: Page 7: 3534-4625
Family educationHealth workers with a high level of PSM explained howfamily education and childhood experiences contributedto their high sense of civic duty and a high orientationto civic participation. Parent modelling and educationled them to serve others and to act for the commongood from a non-self-interested perspective.“I do not take bribes. I have an ideal about the role ofdoctors in society. Their role is not limited to being acare provider at the hospital. They should get out tothe community, sensitize the population during healtheducation sessions. I feel satisfied when we organize around table with practitioners and localrepresentatives, when we organize medical caravans,when we circumcise children for the sake of God. Ilearned these principles through my parents’education. When we were young, we were educated tohelp people, to help others, neighbours, friends,siblings and family members. I cannot explain thesethings, I do not know if it is genetics, but we learnthat we do not live alone but in a society. We dependon each other, we belong to a society, we live with
Title: belrhiti2019 (3).pdf
Doc Creator: djibrine.diallo

Doc Date: 6/2/2024
Codes Applied: Family education and religious beliefs
Excerpt Creator: djibrine.diallo
Excerpt Created On: 6/5/2024
Excerpt Range: Page 8: 4058-4227
Our analysis indicates that religious beliefs may underliepublic service motivation to the extent that these beliefscontribute to an altruistic and compassionate attitude
Title: belrhiti2020 (3).pdf
Doc Creator: djibrine.diallo
Doc Date: 6/2/2024
Codes Applied: Lack of resources
Excerpt Creator: djibrine.diallo
Excerpt Created On: 6/5/2024
Excerpt Range: Page 11: 438-1323
Staff said they were suffering from psychological distress due to poor working conditions, and experienced feel-ings of guilt because of their inability to perform their job adequately and to ease their patients' suffering. Lack of opportunities to experience positive patients outcomes reduced their public service motivation.When you do not have necessary material you are in trouble! It is not only a constraint but a source of suf-fering. Instead of relieving patients' distress, it is us who get stressed. (SMBA 45, doctor)Here, ressources are limited compared to the teach-ing hospital where we were trained. Real world prac-tices are really different. When we first were assigned to this hospital we could not change things around. This is really depressing. We have the ability to pro-vide specialised care but we do not have the necessary ressources to do it! (SMBA 42, doctor)
Title: belrhiti2020 (3).pdf
Doc Creator: djibrine.diallo
Doc Date: 6/2/2024
Codes Applied: Importance of the participation in "decision-making"
Excerpt Creator: djibrine.diallo
Excerpt Created On: 6/5/2024
Excerpt Range: Page 8: 1065-1381
Participative decision- making was perceived by staff as congruent with their professional identity and their public service values. It enhanced their self- esteem and satisfied their needs for autonomy and relatedness and increased their public service motivation. It also increased their perceived autonomy support.
Title: belrhiti2020 (3).pdf
Doc Creator: djibrine.diallo
Doc Date: 6/2/2024
Codes Applied: Lack of resources
Excerpt Creator: djibrine.diallo
Excerpt Created On: 6/5/2024
Excerpt Range: Page 11: 1324-2763
This impacted negatively on their perceived organisa-tional support. This led to crowding out of their public service motivation and lowered their organisational commitment and their well- being.It is really depressing. I do not want to work anymore because I do not have the necessary ressources.[…] I often cry when I watch newborns suffering from intra-muscular injections because nurses are not skilled to administrater intraveineus infusions to newborns and often use instead intramuscular injection for 10 days. I am not only frustrated, I hate entering neonatology service!!!. I only grudgingly go see my patients where-as in the past I loved providing neonatology care. I cannot stand seeing newborn almost dying of hypo-glycaemia 0.3 g/L because they are not adequately fed. This is due to the acute shortage of nurses (one nurse per shift) who are unable to reconciliate be-tween administering antibiotics and treating infec-tions and baby feeding. I am not anymore motivated to cure newborns 'infections but I am terribly stressed avoiding newborns to die from hypoglycaemia. If ba-bies are left alone with the feeding bottles they may die by suffocation. How can we come motivated to work in the next morning? of course not!!! (SMBA 42, paediatrician)Shortage of material reduced their ability to properly care for patients, which reduced their PSM and contrib-uted to a reduced sense of competency, self- efficacy and autonomy.
Title: belrhiti2020 (3).pdf
Doc Creator: djibrine.diallo
Doc Date: 6/2/2024
Codes Applied: Lack of autonomy
Excerpt Creator: djibrine.diallo
Excerpt Created On: 6/5/2024
Excerpt Range: Page 8: 1959-2254
Our analysis showed that the transactional leadership of CEO 1 did not address the basic psychological needs of the staff and specifically the need for autonomy. This not only contributed to low organisational commitment and reduced public service motivation but also to tensions with the unions.
Title: belrhiti2020 (3).pdf
Doc Creator: djibrine.diallo
Doc Date: 6/2/2024

Codes Applied: Importance of the participation in "decision-making"
Excerpt Creator: djibrine.diallo
Excerpt Created On: 6/5/2024
Excerpt Range: Page 8: 2710-3425
CEO 2 also stimulated the emergence of distributed leadership to lower levels of the organisation, which increased trust between the staff and the CEO, and reduced resistance to change. This was considered by mid- level managers as crucial in maintaining the 'public service motivation' of staff, in particular given the perceived limited decision spaces they have over their personal work. We saw that not only senior managers but also mid- level managers engaged in distributing lead-ership. For the latter, participating in decision-making increased their perceived leader support and satisfac-tion of their autonomy needs. This has enhanced their autonomous motivation (intrinsic and public service motivation).
Title: belrhiti2020 (3).pdf
Doc Creator: djibrine.diallo
Doc Date: 6/2/2024
Codes Applied: Lack of autonomy
Excerpt Creator: djibrine.diallo
Excerpt Created On: 6/5/2024
Excerpt Range: Page 7: 3438-4385
The perspective of staffLeadership styleOur analysis shows that the staff found that the transac-tional leadership style of CEO 1 was incongruent with their professional values and their need for autonomy. This contributed to mistrust in the management team, low organisational commitment and a high level of tension with unions.CEO 1, with whom I worked, was authoritative. This was not congruent with my values. I value participa-tive decision making. I try to share with others, I need to be treated the same way by my superior. CEO 1 was just commanding: 'Do this, give this to this person'. I would have accepted and engaged with him if he would have involved me in participative decision mak-ing with other members of the hospital committee, if he would have used polite inquiries, like "Would it be possible to do this?, rather than giving orders without listening to team members or involving them in deci-sion making. (EJMH 25, pharmacist)
Title: belrhiti2020 (3).pdf
Doc Creator: djibrine.diallo
Doc Date: 6/2/2024
Codes Applied: Importance of the participation in "decision-making"
Excerpt Creator: djibrine.diallo
Excerpt Created On: 6/5/2024
Excerpt Range: Page 7: 4386-4552
The participative decision- making style of CEO 2 and his consideration for individuals restored trust in the management team and reduced the tensions with the unions.
Title: Prytherch 2012.pdf
Doc Creator: djibrine.diallo
Doc Date: 6/2/2024
Codes Applied: Community appreciation
Excerpt Creator: djibrine.diallo
Excerpt Created On: 6/8/2024
Excerpt Range: Page 7: 780-980
Community appreciation was identified as being extremely encouraging and the most important source of appreciation for MNH providers by all the respondents, with the exception of those at policy level.
Title: Prytherch 2012.pdf
Doc Creator: djibrine.diallo
Doc Date: 6/2/2024
Codes Applied: Lack of resources
Excerpt Creator: djibrine.diallo
Excerpt Created On: 6/8/2024
Excerpt Range: Page 7: 2746-3133
We are expected to work without gloves when the supplies dry up. Imagine you have to take a delivery and will come in contact with blood and stool. There is a risk of infection. I ask myself will they pay me if I get a problem, and who will take care of my children? When you think of that you won't do your work as its supposed to be done. (Public Health Nurse, female, public facility)
Title: Prytherch 2012.pdf
Doc Creator: djibrine.diallo
Doc Date: 6/2/2024
Codes Applied: Poor Working environment and condition
Excerpt Creator: djibrine.diallo
Excerpt Created On: 6/8/2024
Excerpt Range: Page 7: 3196-3499
The district people delayed in sending the car for so long that the woman died. I was with her, trying to help but I do not have the ability to perform a caesarean section. This caused me much distress and for a long time I didn't want to work anymore. (Enrolled

Nurse Midwife, female, public facility)
Title: Prytherch 2012.pdf
Doc Creator: djibrine.diallo
Doc Date: 6/2/2024
Codes Applied: Poor Working environment and condition Workloads
Excerpt Creator: djibrine.diallo
Excerpt Created On: 6/8/2024
Excerpt Range: Page 8: 3200-3433
My working conditions are very difficult and sometimes I get so tired I feel dizzy. If there were more of us then everything could be better because we would be able to work in shifts. (Enrolled Nurse Midwife, female, public facility)
Title: Prytherch 2012.pdf
Doc Creator: djibrine.diallo
Doc Date: 6/2/2024
Codes Applied: Workloads Lack of training
Excerpt Creator: djibrine.diallo
Excerpt Created On: 6/8/2024
Excerpt Range: Page 10: 3606-3969
Performance and quality of care have been shown to be undermined by a lack of staff, staff assuming multiple roles12 and gap-filling with lower-level staff. The present situation is compounded by the lack of skills-based training and the particular difficulties that lower cadres face in accessing training at all, as also observed in other research in Tanzania32.
Title: ojakaa2014 (1).pdf
Doc Creator: djibrine.diallo
Doc Date: 6/2/2024
Codes Applied: Job satisfaction
Excerpt Creator: djibrine.diallo
Excerpt Created On: 6/7/2024
Excerpt Range: Page 10: 2903-4223
The findings from this study show that levels of satisfac-tion differ on some attributes between the three regionson the one hand, and on the other between public andprivate health facilities. Staff shortages, transportation,inadequate supportive supervision, and a lack of essentialsupplies and functional equipment are notable factors ofthe working environment that contribute to dissatisfac-tion. Allowances (hardship, marriage, over-time), rest andrecuperation are also important compensation aspectsthat would affect satisfaction. This study identified ad-equate training, job security, salary, and supervisory sup-port as critical satisfaction factors, as others have alsoidentified in other settings [8,19,23]. Additionally, thefinding in this study that having a manageable workloadwas a significant contributor to job satisfaction, which isrelated to motivation, supports existing literature [27]. Ahigh proportion of health workers in Turkana feel thattheir workload is not manageable. This could be related togreater staff shortages in Turkana, as observed in our find-ings. Health workers in these settings also reported totaking on additional duties due to inadequate human re-source capacity [28]. This presents an additional problemof workers completing tasks for which they lack skills andare not trained.
Title: ojakaa2014 (1).pdf
Doc Creator: djibrine.diallo
Doc Date: 6/2/2024
Codes Applied: Poor Working environment and condition
Excerpt Creator: djibrine.diallo
Excerpt Created On: 6/7/2024
Excerpt Range: Page 10: 4224-4869
Work environment, remuneration, compensationand retentionInadequate access to electricity, equipment and trans-portation was found to be most critical in Turkana, asexpected. Lack of housing, inadequate payment of sup-port staff, and poor physical state of the health facilitycontribute to a non-conducive working environment.More than 30% of all health workers surveyed did notfeel that they had job security. The working environment inprivate facilities was rated higher than that in Governmentfacilities. Inadequate working conditions, coupled withlow job satisfaction and stability, are bound to demotivatehealth workers and impact retention
Title: songstad2011 (1).pdf
Doc Creator: djibrine.diallo
Doc Date: 6/2/2024
Codes Applied: Inequalities in allowance distribution as a demotivating factor
Excerpt Creator: djibrine.diallo
Excerpt Created On: 6/8/2024
Excerpt Range: Page 8: 0-167
irrespective of their work specialisation. The selec-tion for seminars is biased and causes a lot of mis-understandings and demoralises the workers. (AMO,hospital, FGD)
Title: songstad2011 (1).pdf
Doc Creator: djibrine.diallo
Doc Date: 6/2/2024
Codes Applied: Promotional delays as a demotivating factor

Excerpt Creator: djibrine.diallo
Excerpt Created On: 6/8/2024
Excerpt Range: Page 7: 143-551
I am approaching retirement age, I only have fouryears left at work, and I will probably have a poorpension. Promotion is not handled properly, andthere is no one in the district administration whocares about our promotion.and furthermore:If you follow up on promotion you will waste yourtime. You will run around in the offices withoutresults. This is very discouraging and demoralising.(CO, dispensary, IDI)
Title: songstad2011 (1).pdf
Doc Creator: djibrine.diallo
Doc Date: 6/2/2024
Codes Applied: Poor Working environment and condition
Excerpt Creator: djibrine.diallo
Excerpt Created On: 6/8/2024
Excerpt Range: Page 6: 197-476
Working as a nurse is a vocation, but we have diffi-cult working conditions and a risky working envir-onment. You may be helping an HIV-positivewoman during delivery. Your own life is at risk, andyet the payment is so small, making you unhappyand disturbed. (Nurse, hospital, FGD)
Title: songstad2011 (1).pdf
Doc Creator: djibrine.diallo
Doc Date: 6/2/2024
Codes Applied: Lack of recognition
Excerpt Creator: djibrine.diallo
Excerpt Created On: 6/8/2024
Excerpt Range: Page 7: 1666-1993
I think I am not fairly paid, because right now I have38 years of work experience, but I get the same payas young employees. I have a very long work experi-ence. Others are recently employed and have nowork experience. I have taught them how to workbut they have the same or even higher salary than Ihave. (Nurse, hospital, FGD)
Title: songstad2011 (1).pdf
Doc Creator: djibrine.diallo
Doc Date: 6/2/2024
Codes Applied: Lack of recognition Poor salary
Excerpt Creator: djibrine.diallo
Excerpt Created On: 6/8/2024
Excerpt Range: Page 6: 2648-2974
An employee at the district council gets a very highsalary and plenty of allowances. Their work onlyinvolves paperwork, simply handling paper. Whilewe, the nurses, caring for the souls of human beingsand saving the lives of children, are paid less. This issomething the government has not considered well.(Nurse, hospital, FGD)
Title: Pryterch 2013.pdf
Doc Creator: djibrine.diallo
Doc Date: 6/2/2024
Codes Applied: Gender norm
Excerpt Creator: djibrine.diallo
Excerpt Created On: 6/8/2024
Excerpt Range: Page 14: 124-405
In Burkina Faso, provider sex appeared to play a role inrural staff recruitment and available female providers maybe carrying a particularly high MNH workload. This under-lines the importance of gender-sensitive health workforceplanning and retention strategies for progress in MNH.
Title: Pryterch 2013.pdf
Doc Creator: djibrine.diallo
Doc Date: 6/2/2024
Excerpt Creator: djibrine.diallo
Excerpt Created On: 6/8/2024
Excerpt Range: Page 10: 1226-2001
Regarding the rural workforce, the recruitment of fe-male MNH providers emerged as a specific problem inthis area of Burkina Faso, as has also been found inother conservative settings [26]. Reasons are likely to in-clude the general challenges women face to do well atschool or to forge a career in Burkinabe society [27]. Inaddition, cultural and security concerns appeared to sur-round the posting of single women away from home, orexpecting a husband to relocate for his wife. The com-paratively low numbers of female MNH providers islikely to play a role in the low reported use of somehealth centres. A review of 41 Demographic and HealthSurveys revealed that one quarter of women cited theabsence of a female health provider as a reason not todeliver at a facility [28].
Title: Pryterch 2013.pdf
Doc Creator: djibrine.diallo
Doc Date: 6/2/2024

Codes Applied: Workloads
Excerpt Creator: djibrine.diallo
Excerpt Created On: 6/8/2024
Excerpt Range: Page 11: 3046-4120
Across all the responses, MNH workloads were felt tobe especially heavy. In general, this was attributed to thenature of the work and the overall shortage of staff. InGhana this stood at odds with the perception of urbanproviders that their counterparts in rural areas were lazy -a finding which could again reflect the lack of statusaccorded to rural health work. Lack of staff at facility levelis known to be discouraging and to rob providers ofprofessional exchange and support [39]. In addition,many observed MNH to be an area of occupationalrisk, with efforts to counter this appreciated [40].There were indications from Burkina Faso that otherproviders were reluctant to become too involved in MNHtasks. Despite their low numbers female providers at pri-mary care level in Burkina Faso may carry a disproportion-ate burden of MNH work, as has been observed in othersettings [41]. This could be addressed by revisiting the div-ision of labour across facility teams, with particular atten-tion paid to gender aspects. There could be a need tofurther incentivise MNH work.
Title: Hopwood et al 2023.pdf
Doc Creator: djibrine.diallo
Doc Date: 6/2/2024
Codes Applied: Lack of support and supervision
Excerpt Creator: djibrine.diallo
Excerpt Created On: 6/4/2024
Excerpt Range: Page 5: 98-1102
The participants also highlighted that as a result o□ not being supervised, they □nd it difcult to take work seriously. Some o□ the partici-pants explained:Our caseload in some districts is very high in a way that □or us to even manage ourselves is difcult, and more also the caseload and work is plenty □or us. This has made us psychologically out o□ tract, because o□ caseload, no motivation, no encouragement and no one is checking us to know what is happening … To let a single mental nurse cover a whole district is not easy thing, and no one cares about you, even i□ you are sick, they don’t care … No one is coming out o□ the Minis-try to check on us. When we hear supervisors are com-ing, we too will take our work seriously. We take our supervisors to our success cases… (KII Participant 4)Also lack o□ expanding the current □acility beyond Freetown; we only have one mental home in Freetown and the service needs to be extended to the provinces at least at district levels… (KII Participant 10)L
Title: Hopwood et al 2023.pdf
Doc Creator: djibrine.diallo
Doc Date: 6/2/2024
Codes Applied: Lack of training Lack of recognition
Excerpt Creator: djibrine.diallo
Excerpt Created On: 6/4/2024
Excerpt Range: Page 4: 197-796
Am also appealing that some o□ us are on the pathway now actually to retirement, so we are encouraging and appealing to the Ministry o□ Health and Sanitation to continue training more mental health nurses in the country. Because we are about eighteen or nineteen remaining: two died. (KII Participant 7)Nurses in certain districts reported high caseload as a challenge which o□ten a□ects their work activities. Nurses also believe that they would be taken more seriously i□ they were greater in number. Furthermore, nurses reported poor motivation. This was o□ten attributed to a lack o□ recognition
Title: Hopwood et al 2023.pdf
Doc Creator: djibrine.diallo
Doc Date: 6/2/2024
Codes Applied: Low priority of the mental health service
Excerpt Creator: djibrine.diallo
Excerpt Created On: 6/4/2024
Excerpt Range: Page 6: 1080-1290
Mental health service delivery is o□ten stigmatised. As a result, mental health services are o□ten given low priority compared to other health services. This is o□ten a source o□ discouragement □or these nurses.
Title: Hopwood et al 2023.pdf
Doc Creator: djibrine.diallo
Doc Date: 6/2/2024
Codes Applied: Lack of resources
Excerpt Creator: djibrine.diallo
Excerpt Created On: 6/4/2024
Excerpt Range: Page 6: 2177-2409
Finally, lack o□ resources was identi□ed as a key barrier to success□ul implementation o□ the nurse-led mental health services. Lack o□ resources included issues with outdated policies, poor salaries and lack o□ access to medication.
Title: Hopwood et al 2023.pdf
Doc Creator: djibrine.diallo
Doc Date: 6/2/2024
Codes Applied: Lack of policies supporting mental health service

Excerpt Creator: djibrine.diallo
Excerpt Created On: 6/4/2024
Excerpt Range: Page 6: 2428-3908
ierra Leone continues to use the Lunacy Act o□ 1902 □or its mental health legislation. This legal □ramework is outdated and does not address the current challenges □aced in mental healthcare. In 2012, the □rst ever national Mental Health Policy was launched in Sierra Leone (Bah et al., 2018). At the time o□ the participant interviews, this policy was being updated. Some o□ the participants raised concerns regarding creating new policies and strategies as well as ensuring these policies are converted into legislation and enacted. It was also recommended that these policies should incorporate the current needs o□ mental healthcare in Sierra Leone such as in□rastructure and sa□ety.We need a very robust service policy, am sorry because with policy, i□ this policy are enacted then mental health service will be high up, there will be at par with other services … but we need a very good policy in terms o□ emulation, in terms o□ motivation, in terms o□ conditions and development, in terms o□ encourage other sta□s □rom other disciplines to come and work in the hospital. (KII Participant 2)But sometimes it comes to implementation, you know the Ministry or sta□ would have sat down and come up with very good strategy but yet we need □unding to promote or to implement. And i□ not, then what hap-pens, you know things haven't worked, you know it is just support, you know in implementation because really it is the key □or most o□ the things to be done. (KII Participant 9)P
Title: Hopwood et al 2023.pdf
Doc Creator: djibrine.diallo
Doc Date: 6/2/2024
Codes Applied: Poor salary
Excerpt Creator: djibrine.diallo
Excerpt Created On: 6/4/2024
Excerpt Range: Page 6: 3922-4916
t was identi□ed that mental health nurses are not well paid and that this additionally contributes to their poor motiva-tion. The 19 nurses completed either the diploma or cer-ti□cate in mental health nursing in 2013, but many o□ them have not received any □nancial increment in their salary to refect their additional quali□cations. One or two are not being paid due to reasons related to problems within the Government and central HR processes. Nurses must o□ten □und their phone credit, printing and petrol themselves. Sub-sequently, other healthcare pro□essionals are unwilling to join the mental health work□orce because o□ the poor sala-ries. One o□ the participants stated:I thank you so much □or that important question. I□ I was in place to promote mental health in this country, I think □oremost o□ all this is a no go area □or our other colleagues in the medical pro□ession because they don't want to go there because there is no money and there is no motivation. (KII Participant 7)
Title: Hopwood et al 2023.pdf
Doc Creator: djibrine.diallo
Doc Date: 6/2/2024
Codes Applied: Passion as a driven force for intrinsic motivation
Excerpt Creator: djibrine.diallo
Excerpt Created On: 6/2/2024
Excerpt Range: Page 9: 409-1054
PassionNurses continue to provide services despite the challenges they experience in providing mental health services. This is as a result o□ the passion □or the job. As such, passion is a driving □orce and a key □acilitator to nurse-led mental health service in Sierra Leone. One participant stated:Just that I have passion □or the job and the patients that I am treating; i□ not I should have given up □rom this mental health work long since. (FGD Participant 6)I am improving my everyday work □rst o□ all, I like what am doing, I am passionate o□ what am doing, and everyday am expectant that I am going to see my patients. (KII Participant 3)
Title: Hopwood et al 2023.pdf
Doc Creator: djibrine.diallo
Doc Date: 6/2/2024
Codes Applied: Power distance and influence
Excerpt Creator: djibrine.diallo
Excerpt Created On: 6/2/2024
Excerpt Range: Page 7: 1842-2088
One o□ the participants explained that power and in□uence may have an impact on service delivery since approval □or certain resources including medications o□ten takes time because it must go through di□erent phases o□ approval at management level
Title: Hopwood et al 2023.pdf
Doc Creator: djibrine.diallo
Doc Date: 6/2/2024
Codes Applied: continuous professional development opportunities
Excerpt Creator: djibrine.diallo
Excerpt Created On: 6/2/2024
Excerpt Range: Page 8: 2075-2191
Mental health nurses can be motivated to improve their skills and participate in continu-ous pro□essional development
Title: Hopwood et al 2023.pdf
Doc Creator: djibrine.diallo
Doc Date: 6/2/2024

Codes Applied: Policies supporting
Excerpt Creator: djibrine.diallo
Excerpt Created On: 6/2/2024
Excerpt Range: Page 8: 2219-2305
also be motivated through policies that support the efcient mental healthcare services.
Title: Hopwood et al 2023.pdf
Doc Creator: djibrine.diallo
Doc Date: 6/2/2024
Codes Applied: Uncertainty avoidance
Excerpt Creator: djibrine.diallo
Excerpt Created On: 6/2/2024
Excerpt Range: Page 7: 3046-3475
I□ I want any utensils, drugs or whatever, the chain o□ command is □or the person responsible to write through the ofcial channel through the psychiatrist in charge and then it goes to the responsible person in the Ministry, the HQ and then □rom there we can then be supplied, sometimes we are not, sometimes it takes quite some time, sometimes the availability is a problem to the healthcare delivery service. (KII Participant 2)
Title: brenya2016.pdf
Doc Creator: djibrine.diallo
Doc Date: 6/2/2024
Codes Applied: Poor Working environment and condition
Excerpt Creator: djibrine.diallo
Excerpt Created On: 6/6/2024
Excerpt Range: Page 11: 3335-4039
Although civil servantshave compassion, civic duty to society, self-sacrifice,and commitment to public interest, they are not willingto continuously work within the public sector. In otherwords, though civil servants recognize and have the zealto serve societal interest, they are reluctant to eternallypursue such interests within the public sector. Thisproblem can be attributed to poor working conditionsin various agencies within the public sector. We there-fore emphasize that if working conditions are betteredwithin the public sector, civil servants will surely com-mit their future to the sector and continually pursuesocietal interests since their motives are grounded onpursuing public interest.
Title: gould-williams2015 (2).pdf
Doc Creator: djibrine.diallo
Doc Date: 6/2/2024
Excerpt Creator: djibrine.diallo
Excerpt Created On: 6/6/2024
Excerpt Range: Page 4: 296-679
However, the "con-trarian position" is not consistent with PSM theory, which suggests that PSM begins to develop prior to joining the organization and hence might be managed through appropriate recruitment and selection practices. For example, Perry and Wise (1990) highlight the importance o□ sociohistorical □actors, including parental values, edu-cation, and religious a□□liation.
Title: gould-williams2015 (2).pdf
Doc Creator: djibrine.diallo
Doc Date: 6/2/2024
Codes Applied: Knowledge gap
Excerpt Creator: djibrine.diallo
Excerpt Created On: 6/7/2024
Excerpt Range: Page 19: 381-530
Assessing the e□□ects o□ employees' PSM, per□ormance expectations, and perceived abilities on their well-being is a promising area □or □uture research
Title: gould-williams2015 (2).pdf
Doc Creator: djibrine.diallo
Doc Date: 6/2/2024
Codes Applied: Knowledge gap
Excerpt Creator: djibrine.diallo
Excerpt Created On: 6/7/2024
Excerpt Range: Page 19: 810-1576
As noted earlier, the Egyptian culture is highly col-lectivistic, with Islamic work values playing a signifcant role in society. Since many o□ these values are similar to those o□ PSM, it is possible that Islamic work values also a□□ected our respondents' behaviors. We await □uture studies to disentangle the unique contributions o□ PSM vis-à-vis collectivism, which appears to have synergistic e□□ects in the Egyptian public sector. Also, it is possible □or respondents to di□□er in the extent to which they endorse collectivistic values. This in turn may a□□ect the strength o□ the relationships in our model (PSM to P-O ft; P-O ft to employee outcomes). Ho□stede's (1980) cultural dimensions □ramework could prove a use□ul lens through which to view these issues.
Title: gould-williams2015 (2).pdf
Doc Creator: djibrine.diallo

Doc Date: 6/2/2024
Excerpt Creator: djibrine.diallo
Excerpt Created On: 6/7/2024
Excerpt Range: Page 17: 955-1208
Our research presents several noteworthy □ndings. First, the positive e□□ects o□ PSM on employee outcomes are contingent on the degree o□ congruence between employees’ values and those o□ the organization (Bright 2008; Kim 2012; Wright and Pandey 2008).
Title: gould-williams2015 (2).pdf
Doc Creator: djibrine.diallo
Doc Date: 6/2/2024
Excerpt Creator: djibrine.diallo
Excerpt Created On: 6/6/2024
Excerpt Range: Page 4: 2794-3084
PSM theorists argue that employees with high PSM are prepared to sacri□ce personal gains to help the community (Perry and Wise 1990). Thus, employees with high PSM should be desirous o□ serving the community and willing to help individu-als within the organization to achieve its objectives.
Title: Sussana Adjei-Mensah 2023.pdf
Doc Creator: djibrine.diallo
Doc Date: 6/2/2024
Excerpt Creator: djibrine.diallo
Excerpt Created On: 6/4/2024
Excerpt Range: Page 12: 1104-2279
The study concludes that due to government inaction on the conditions of service of health workers such as low remuneration, inadequate opportunities for staff development, poor equipment replacement policies, political interferences in their workplaces, excessively high standards of living and inadequate housing and personal reasons that include the need to improve the educational status, family and friends influence, career advancement and the need to achieve future ambitions, Ghana is losing the majority of her finest health workers to the developed nations each year. To this effect, the migration of health care professionals from developing to developed nations has negative effects on the delivery of health care in Ghana. Since the government is unable to deal with the push and pull economic factors to address the fundamental health workers needs, such as remuneration issues, career development, hospital infrastructure, resource availability, hospital management, political issues, family issues, and training, education, and development and competition from the private sector, many health workers are forced to seek "greener pastures" outside the country.
Title: Sussana Adjei-Mensah 2023.pdf
Doc Creator: djibrine.diallo
Doc Date: 6/2/2024
Excerpt Creator: djibrine.diallo
Excerpt Created On: 6/4/2024
Excerpt Range: Page 10: 2169-2859
The study found that the highly ranked factors influencing brain drain among health workers in Ghana include low remuneration even relative to countries within the sub-region, the need to better their education to develop their careers, the need to achieve future ambitions, inadequate opportunities for staff development, poor replacement policies for equipment, inadequate accommodation. The least ranked factors included active canvassing of western countries for health professionals, inadequate consumables for work, too high standards of living, family and friends influence, high political interferences, and the inability to acquire basic social amenities e.g. housing, transport etc
Title: Naima Said Sheikh, 2023.pdf
Doc Creator: djibrine.diallo
Doc Date: 6/2/2024
Codes Applied: Masculinity versus feminity
Excerpt Creator: djibrine.diallo
Excerpt Created On: 6/4/2024
Excerpt Range: Page 9: 339-505
Therefore, the patriarchalculture in Somalia [33] that keeps women subordinate might have negatively affected thefemale gynaecologists’ motivation and job satisfaction
Title: Naima Said Sheikh, 2023.pdf
Doc Creator: djibrine.diallo
Doc Date: 6/2/2024
Codes Applied: Importance of intrinsic motivation "inner motivation"
Excerpt Creator: djibrine.diallo
Excerpt Created On: 6/4/2024
Excerpt Range: Page 9: 614-980
Surprisingly, volunteers have the highest motivation sc0re compared to salaried employees.This implies that the salary is not the greatest motivator for work, but rather that inner motiva-tion influences health workers’ motivation. This suggests that constructs concerning innermotivation are of more importance than constructs concerning other aspects of motivation.
