# Supplementary material for: How Organisational and Socio-Cultural Contexts Shape Healthcare Workers’ Intrinsic, Prosocial, and Public Service Motivation in Africa: A Scoping Review
Source: Int J Health Policy Manag. 2025 Oct 4;14:8861. doi: 10.34172/ijhpm.8861 (PMC12958157; doi:10.34172/ijhpm.8861)
Supplement: Supplementary file 9 — contains Table S6. [file ijhpm-14-8861-s009.pdf]

Article title: How Organisational and Socio-Cultural Contexts Shape Healthcare Workers' Intrinsic, Prosocial, and Public Service Motivation in Africa: A Scoping Review

Journal name: International Journal of Health Policy and Management (IJHPM)

Authors' information: Djibrine Diallo[1,2]*, Bruno Marchal[3], Zakaria Belrhiti[1,2]

[1]Mohammed VI International School of Public Health, Mohammed VI University of Sciences and Health, Casablanca, Morocco.

[2]Laboratory of Public Health and Management Department, Mohammed VI Center for Research & Innovation, Rabat, Morocco.

[3]Department of Public Health, Institute of Tropical Medicine, Antwerp, Belgium.

***Correspondence to:** Djibrine Diallo; Email: ddiallo@um6ss.ma

Citation: Diallo D, Marchal B, Belrhiti Z. How organisational and socio-cultural contexts shape healthcare workers' intrinsic, prosocial, and public service motivation in Africa: a scoping review. Int J Health Policy Manag. 2025;14:8861. doi:10.34172/ijhpm.8861

Supplementary file 9

Author (Year)	Method	Tool/Instrument Used	Motivation Type	Psychometric Info (e.g., Validity/Reliability)	Notes
Adjei-Mensah (2023)	Quantitative	Survey scale	IM	Cronbach's alpha > 0.7	National survey
Sheikh et al. (2023)	Quantitative	Structured questionnaire	IM,	No detailed validity data reported	Includes gender-focused results
Rim et al. (2021)	Quantitative	Standard questionnaire	IM	Reliability score reported	Small sample size
Gould-Williams et al. (2015)	Quantitative	Perry's PSM scale (adapted)	PSM, PM	Validated; strong psychometric properties	Cross-national comparison
Ojakaa et al. (2014)	Quantitative	Questionnaire including IM/PSM proxies	IM, PM	Limited psychometric reporting	Focus on rural Kenya (factors related to HWs' motivation and the work environment
Brenya et al. (2016)	Mixed-methods	Interview + structured questionnaire	PSM, PM	Not reported	Cultural norms analysis
Luboga et al.	Mixed-	Survey +	IM, PM	No detailed	District-level

(2011)	methods	interview		psychometric info	data (factors related to HWs' motivation)
Qualitative studies (n=11)	Qualitative	Interviews, FGDs	Thematic analysis	N/A – no psychometric tools	See ICAMO synthesis
